# Deep active learning for classifying cancer pathology reports

**DOI:** 10.1186/s12859-021-04047-1

**Published:** 2021-03-09

**Authors:** Kevin De Angeli, Shang Gao, Mohammed Alawad, Hong-Jun Yoon, Noah Schaefferkoetter, Xiao-Cheng Wu, Eric B. Durbin, Jennifer Doherty, Antoinette Stroup, Linda Coyle, Lynne Penberthy, Georgia Tourassi

**Affiliations:** 1 Oak Ridge National Lab, Oak Ridge, TN USA; 2 The Bredesen Center, The University of Tennessee, Knoxville, TN US; 3 Louisiana Tumor Registry, Louisiana State University Health Sciences Center, School of Public Health, New Orleans, LA USA; 4 College of Medicine, University of Kentucky, Lexington, KY USA; 5 Utah Cancer Registry, University of Utah School of Medicine, Salt Lake City, UT USA; 6 New Jersey State Cancer Registry, New Jersey Department of Health, Trenton, NJ USA; 7 Information Management Services Inc., Calverton, MD USA; 8 Surveillance Research Program, Division of Cancer Control and Population Sciences, National Cancer Institute, Bethesda, MD USA

**Keywords:** Active learning, Cancer pathology reports, Text classification, Deep learning, Convolutional neural networks

## Abstract

**Background:**

Automated text classification has many important applications in the clinical setting; however, obtaining labelled data for training machine learning and deep learning models is often difficult and expensive. Active learning techniques may mitigate this challenge by reducing the amount of labelled data required to effectively train a model. In this study, we analyze the effectiveness of 11 active learning algorithms on classifying subsite and histology from cancer pathology reports using a Convolutional Neural Network as the text classification model.

**Results:**

We compare the performance of each active learning strategy using two differently sized datasets and two different classification tasks. Our results show that on all tasks and dataset sizes, all active learning strategies except diversity-sampling strategies outperformed random sampling, i.e., no active learning. On our large dataset (15K initial labelled samples, adding 15K additional labelled samples each iteration of active learning), there was no clear winner between the different active learning strategies. On our small dataset (1K initial labelled samples, adding 1K additional labelled samples each iteration of active learning), marginal and ratio uncertainty sampling performed better than all other active learning techniques. We found that compared to random sampling, active learning strongly helps performance on rare classes by focusing on underrepresented classes.

**Conclusions:**

Active learning can save annotation cost by helping human annotators efficiently and intelligently select which samples to label. Our results show that a dataset constructed using effective active learning techniques requires less than half the amount of labelled data to achieve the same performance as a dataset constructed using random sampling.

**Supplementary Information:**

The online version supplementary material available at 10.1186/s12859-021-04047-1.

## Background

The latest developments in natural language processing (NLP) have made notable progress in automating classification and information extraction from clinical texts. The current state-of-the-art techniques are generally deep learning (DL) architectures such as Convolutional Neural Networks (CNNs), which have been shown to outperform traditional machine learning techniques and rule-based approaches in clinical text applications [1–3]. However, a common drawback of DL models is that they tend to require a large amount of training data to achieve high performance. This is a significant problem particularly in clinical applications where obtaining gold-standard labels is difficult and subject to constraints.

A key goal of active learning is to maximize the effectiveness of obtaining additional labelled training data for a given machine learning model. This is achieved by using the model itself to actively select the set of unlabelled data that will be most informative to the model if it were labelled. For example, data associated with common classes that the model is already familiar with may be less informative than data from unseen classes or data on which the model has low confidence. Compared to randomly labelling additional data, active learning enables the model to reach higher performance using fewer additional labelled samples, thereby increasing the efficiency and effectiveness of human annotators [4]. This approach is especially useful for applications such as clinical text classification where annotated data is expensive and time-consuming to obtain.

Cancer pathology reports are an important application where active learning can have real-world impact. Cancer is the second leading cause of death in the United States.^1^ As part of its mission, the National Cancer Institute’s (NCI) Surveillance, Epidemiology, and End Results (SEER) program works with population-based cancer registries around the country to collect and publish cancer data including patient demographics, primary tumor site, tumor morphology and stage at diagnosis, first course of treatment, and follow-up for vital status [5]. This information is critical for cancer research and surveillance. While some of this information is stored in structured databases and can be easily extracted, key data elements such as primary tumor site and tumor morphology are generally recorded within unstructured cancer pathology reports that are written during the time of diagnosis. Each year, skilled human Certified Tumor Registrars (CTRs) must manually annotate hundreds of thousands of cancer pathology reports to extract these data elements. Recent research has made major strides toward automating portions of this process [1, 2]; unfortunately, these approaches still do not have the same level of accuracy as human annotators and often have low performance on rare cancer types with few training examples [2, 6]. Active learning can help address these weaknesses by prioritizing expert annotation of pathology reports that maximize the effectiveness of these automated approaches, thus helping close the gap in performance.

While active learning has been effectively applied to a wide variety of applications, including image classification, speech recognition, and natural language parsing, [7–10], its application to text classification on cancer pathology reports and other clinical text is relatively limited. Unlike other text classification tasks, classifying cancer pathology reports presents a distinctive set of challenges. These reports are characterized by the lack of a universal structure, variation in linguistic patterns, and the use of specific jargon. Furthermore, documents can be several pages long, only a few keywords or keyphrases in the document may be relevant to a specific classification task, and there may be long-distance linguistic dependencies across different sections of a document [2]. In addition, certain tasks may contain a large number of classes (i.e. 525 histology types), and the number of documents per class tends to be highly unbalanced.

In this paper, we evaluate the effectiveness of different active learning techniques on the task of classifying cancer pathology reports. We test a wide selection of different active learning strategies that have been successfully applied in other applications. Using a CNN-based approach as our learning model, we focus on two classification tasks—identifying cancer subsite and histology. Our contributions are as follows:

- We perform a detailed comparative evaluation of different active learning techniques on two different text classification tasks using cancer pathology reports.
- We examine and compare the performance of active learning when being applied on two scenarios. In the first set of experiments, we use a large dataset with 15K initial labelled samples, adding 15K additional labelled samples each iteration of active learning. For the second set of experiments, we use a small dataset with 1K initial labelled samples, adding 1K additional labelled samples each iteration.
- Given that clinical text classification tasks generally have high class imbalance, we perform a detailed analysis of how different active learning techniques affect low prevalence classes.
- To our knowledge, this is the first work to perform a critical comparison of different active learning techniques for clinical text classification that utilizes a deep learning model as the classifier.

### Related work

The benefits of classic active learning techniques that incorporate machine learning models have been widely studied for a variety of tasks [4, 11–14]. In the context of NLP, notable work includes Settles et al. [15] who evaluated 15 query strategies for the task of sequence labeling using conditional random fields on eight different corpora. Even though performance of individual active learning strategies varied across different corpus, the best results were obtained with information density, sequence vote entropy, sequence entropy, and least confidence.

Although active learning and deep learning have each been researched extensively, the current literature at the intersection of both focuses mostly on image classification [16]. Wang et al. [17] proposed a framework that combines uncertainty sampling techniques with pseudo-labeling to reduce human annotation in the context of image classification using CNNs; their technique uses softmax confidence thresholds in the decision process. Zhang et al. [18] explored deep active learning with CNNs for text classification by applying an algorithm called Expected Gradient Length. This work is related to our study; however, it has a specific focus on word embeddings and representation learning.

Few studies have explored AL in the specific context of clinical NLP. These existing works are older studies that focus on using traditional machine learning approaches rather than state-of-the-art deep learning models. Chen et al. [19] applied seven active learning algorithms using logistic regression for the task of binary clinical text classification. In a similar work, Kholghi et al. [20] applied least confidence and information density to the task of medical concept extraction, and showed that active learning can help classifiers to reach a target performance score using as little as 23% of the total data available. Figueroa et al. [21] applied three active learning algorithms—distance-based, diversity-based, and a combination of both—on five datasets. They used support vector machines as the base classifier for clinical text classification and concluded that diversity algorithms respond better on datasets with high diversity, and distance algorithms perform better on datasets with low uncertainty.

We extend these previous studies by performing a thorough evaluation of 11 different active learning techniques using a modern CNN model as our classifier. We compare performance on two different clinical text classification tasks—extracting subsite and histology from cancer pathology reports. We show that under certain conditions, some active learning strategies clearly beat out others; we expect that these results may provide a useful starting point for other applications of active learning in the context of clinical text classification.

## Methods

### Active learning

In the active learning scenario, we begin with an initial training set ${\mathcal {L}}_0$ of labeled samples and use it to train a classification model with parameter estimates $\theta _0$. Then, we apply this model on a set of unlabelled data ${\mathcal {U}}_0$ and use a query strategy $\phi (x_i| \theta _0)$ to assign an informativeness measure to each sample $x_i$ in ${\mathcal {U}}_0$; this informativeness measure indicates how helpful that sample would be to the classification model if it were to be trained on that sample. We then obtain ground truth labels for a subset of *n* samples in ${\mathcal {U}}_0$ with the highest informativeness value $\phi (x_i| \theta _0)$. This subset is moved from ${\mathcal {U}}_0$ to ${\mathcal {L}}_0$ to form the new larger training set ${\mathcal {L}}_1$ and new smaller unlabelled set ${\mathcal {U}}_1$.

A new classification model with parameter estimates $\theta _1$ is then trained on ${\mathcal {L}}_1$ and applied to ${\mathcal {U}}_1$. Once again, the query strategy $\phi (x_i| \theta _1)$ is used to select the most informative *n* samples from ${\mathcal {U}}_1$ to label and add to ${\mathcal {L}}_1$ to form ${\mathcal {U}}_2$ and ${\mathcal {L}}_2$. This process is repeated until the classification model attains the desired performance or until no samples remain in ${\mathcal {U}}$.

In the following subsections, we describe the various active learning query strategies that we evaluate in this work.

#### Uncertainty sampling

One of the most common active learning query strategies is *uncertainty sampling* [22], where $\phi$ is calculated based on the prediction confidence of the classifier—the assumption is that the lower the confidence of a given sample, the more informative it will be for the model. Within the uncertainty sampling domain, ***least confidence (LC)*** is a simple algorithm which calculates $\phi$ based on Eq. 1:

1 $$\begin{aligned} \phi ^{LC}(x) = 1 - P(y^*|x;\theta ) \end{aligned}$$

where $y^*$ is the predicted class for a given sample, i.e., the class with the highest softmax value, and $P(y^*|x;\theta )$ represents the softmax value associated with that class.

Another uncertainty based query strategy introduced by Schein et al. [23] is ***marginal sampling (MC)***. Whereas least confidence only considers the highest softmax value from each predicted sample, marginal sampling utilizes the difference in confidence between the two most likely classes for each predicted sample (Eq. 2):

2 $$\begin{aligned} \phi ^{MS}(x) = 1 - (P(y_{1}^*|x;\theta ) - P(y_{2}^*|x;\theta )) \end{aligned}$$

where $y_{1}^*$ and $y_{2}^*$ represents the two classes associated with the highest and second highest softmax values, respectively.

A third uncertainty sampling query strategy, named ***ratio of confidence (RC)*** in this paper, considers the ratio between the top two classes with the highest softmax values (Eq. 3):

3 $$\begin{aligned} \phi ^{RC}(x) = \frac{P(y_{1}^*|x;\theta )}{P(y_{2}^*|x;\theta )} \end{aligned}$$

Finally, Shannon Entropy [24], or ***entropy sampling (ES)***, has also been widely used as an uncertainty-based query strategy. Under this approach, $\phi$ is an entropy-based metric described in Eq. 4:

4 $$\begin{aligned} \phi ^{ES}(x) = \sum _{c=1}^{C} P(y_{i}|x;\theta )* \log P(y_{i}|x;\theta ) \end{aligned}$$

where $\sum _{c=1}^{C}$ represents the summation over all possible classes, and $P(y_{i}|x;\theta )$ is the softmax value associated with class $y_i$. Thus, unlike previous uncertainty sampling techniques, ***ES*** takes into consideration the softmax distribution across all possible classes.

#### Diversity sampling

*Diversity sampling (DS)* algorithms aim to maximize the diversity of the training dataset and calculate $\phi$ based on a similarity measure between the samples in the training set. Traditionally, diversity sampling algorithms were applied to machine learning approaches that utilized fixed-length input vectors such as TF-IDF, and the similarity measures for $\phi$ could be applied directly on these input vectors. However, in the context of deep learning models, the inputs are typically matrices of word embeddings that may or may not be zero-padded.

Therefore, to effectively utilize diversity sampling, we first generate a fixed-length document vector representation for each document on which we can then apply the similarity metric. In our study, these document vectors are the outputs from the penultimate layer of our text CNN model (described in detail in the “TextCNN” section). This vector represents the most important features of each document captured by the convolution filters that are used to make the classification decision for the given task.

We implement two DS algorithms which are named ***Euclidean Cluster-Based Sampling (EC)*** and ***Cosine Cluster-Based Sampling (CC)***. We begin by separating documents in our training set by class and representing the document embeddings for each class as a unique cluster; within a given cluster, we assume that documents closer to the cluster centroid are less informative than documents that are further away from the cluster centroid. Given a sample in the unlabelled set, we calculate $\phi$ based on how far the document embedding is to the nearest cluster centroid. The difference between the algorithms is the metric used: Euclidean distance or cosine similarity. Algorithm 1 describes the implementation details.

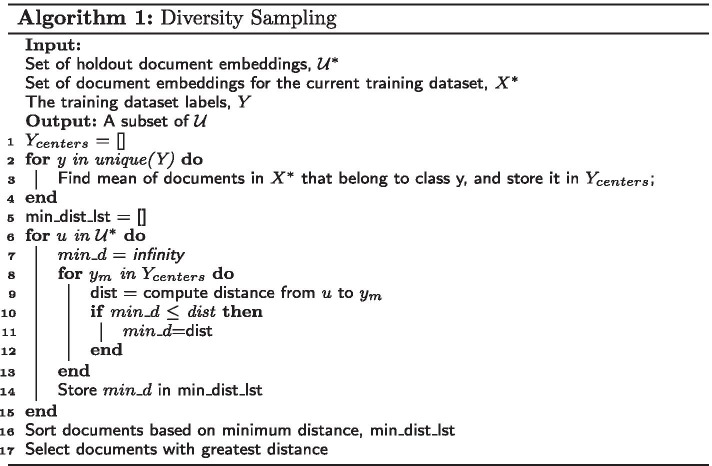



#### Query-by-committee

The core idea behind Query-by-committee (QBC) based active learning [4, 25] is to train multiple predictive models (the committee) and calculate $\phi$ based on the disagreement between the models. The committee makes predictions on the holdout set, and samples are ranked based on how much disagreement there exists within the committee. Samples associated with the highest disagreement are selected and added to the training dataset.

In this work, we utilize a committee of 24 CNNs (described in greater detail in the “TextCNN” section) for all QBC-based methods. Each CNN is independently trained on the training data available during each iteration of active learning. Then, we test three different methods to measure the disagreement between the committee members. In our first method, which is named ***Softmax Sum (SS)***, we average the softmax score vectors from all CNNs in the committee for each document in the holdout set. Then, we apply a method similar to *Least Confidence* and rank the documents based on the maximum softmax score across all possible classes. Documents with the lowest max-softmax values are labelled and moved to the training set. Our implementation is described in Eq. 5:

5 $$\begin{aligned} \phi ^{SS}(x) = 1 - \left(\sum _{h}^{{\mathcal {H}}} P(y^{**}|x;\theta ^h)/{\mathcal {H}}\right) \end{aligned}$$

where $${\mathcal {H}}$$ is the number of members in the committee and $$y^{**}$$ represents the softmax value associated with the class that has the maximum average softmax score across the committee.

For our second method, we apply ***Vote Entropy (VE)***, originally implemented by [26] for the task of part-of-speech tagging. For each document in the holdout set, this method first aggregates the class predictions among the committee members and then utilizes entropy as a measure of disagreement. Our implementation is described in Eq. 6:

6 $$\begin{aligned} \phi ^{VE}(x) = -\sum _c \frac{V(c,x)}{H} \log \frac{V(c,x)}{H} \end{aligned}$$

where *V*(*c*, *x*) represents the number of committee members that predict class *c* for document *x*.

Lastly, we utilize a modified version of Kullback–Leibler (KL) Divergence originally proposed by Pereira et. al. [27], called ***Kullback–Leibler Divergence to the Mean (KL-D)***. KL Divergence is a common method to measure the difference between two probability distributions. In KL-D, we quantify the disagreement within the committee by calculating the mean KL Divergence between each committee member’s softmax vector and the average softmax vector of the whole committee. Our implementation is described in Eq. 7:

7 $$\begin{aligned} \phi ^{KL-D}(x) = \frac{1}{H}\sum _{h}^{{\mathcal {H}}} \sum _{c}^{{\mathcal {C}}} P(y=c|x;\theta ^h) \log \left( \frac{ P(y=c|x;\theta ^h)}{\frac{1}{H}\sum _{h}^{{\mathcal {H}}}P(y=c|x;\theta ^h)}\right) \end{aligned}$$

#### Density-weighted method

Previous work has suggested that methods such as *uncertainty sampling* and QBC are predisposed to select outliers [28]. To solve this issue, Settles and Craven [15] proposed the method of ***Information Density (ID)***. This method accounts for both uncertainty and diversity by weighting the informativeness scores assigned by any uncertainty sampling technique with a similarity term subject to parameter $\beta$. In practice, this method attempts to select samples that the model is uncertain about but that are also similar to other samples in the dataset. Our implementation is shown in Eq. 8:

8 $$\begin{aligned} \phi ^{ID}(x) = \phi ^{uncertainty}(x) * \left( \frac{1}{N}\sum _{n=1}^{N} sim(x,x^n) \right) ^\beta \end{aligned}$$

where *N* represents the total number of samples in the holdout set.

In terms of similarity metrics, the authors of the original paper applied exponential Euclidean distance, KL-divergence, and cosine similarity. They reported the last one to be the most effective. For our implementation, we utilize marginal sampling for $\phi ^{uncertainty}$, cosine similarity for $sim(x,x^u)$, and $\beta =1$. We utilize the softmax vectors from the CNN to calculate $\phi ^{uncertainty}$ and the document embeddings generated by the penultimate layer of the CNN to calculate $sim(x,x^u)$.

#### Meta learning

We propose a novel active learning strategy which consists of training a separate machine learning algorithm to predict which samples will be most informative to the base CNN classifier. The intuition is that if we are able to predict what documents will be misclassified by our model based on the confidence scores generated by the CNN, then we could query those samples and add them to our training dataset.

To achieve this, we first create a new training meta-dataset which consists of the logit vectors ($\vec{x}^{meta}$) obtained by running the trained CNN model on the validation and test datasets used for active learning. This new dataset also contains a binary label ($y^{meta}$) that represents whether the CNN correctly classified the corresponding ground truth labels. Then, we use this meta-dataset to train a separate random forest classifier with 100 trees; this random forest learns how likely the CNN will misclassify a given document based on the CNN’s relative confidence across the possible classes. For each document in the holdout set, we obtain the CNN’s logit vectors and then calculate $\phi$ based on the random forest’s confidence that the CNN will misclassify that document (number of individuals trees out of 100). We refer to this method as ***Meta Learning (ML)***. To the best of our knowledge, we have not seen an implementation of this technique in the current literature.

### Application to cancer pathology reports

Cancer pathology reports are a critical resource for cancer surveillance and research. A cancer pathology report is a medical document written by a pathologist that records the cancer diagnosis of cells and tissues examined under a microscope. Cancer pathology reports are generally multi-page documents with highly technical language and contain a variety of detailed information, including but not limited to patient information, specimen details, descriptions of the sample as seen by the naked eye and under a microscope, cancer diagnosis, pathologist and laboratory information, and additional comments. While we are unable to share specific examples of pathology reports from our experimental dataset due to privacy restrictions, example pathology reports can easily be found online.

As part of its mission, the NCI SEER program collects hundreds of thousands of cancer pathology reports each year in partnership with cancer registries around the United States. Human experts must then manually annotate these reports for key data elements related to cancer primary site and morphology. To help ease the burden on human annotators, previous work has applied deep learning techniques such as CNNs to automatically extract these key data elements. In these studies, a dataset of cancer pathology reports is matched with gold standard human-annotations for key data elements, such as site, subsite, histology, and behavior. A machine learning model is then trained to predict these key data elements—this is generally treated as a single-task or multi-task document classification problem where the input is a cancer pathology report and the output is one or more data elements [1, 2]. However, existing methods still do not achieve high enough accuracy to fully replace human annotators, especially on cancer types with low prevalence and few training examples [2, 6].

Active learning can help address this gap in performance by identifying pathology reports that are especially difficult for automated methods so that human annotators can prioritize annotation of these reports. This has the potential to improve the performance of these automated methods more than if humans experts annotated a random selection of additional cancer pathology reports. To better understand the potential benefits of active learning, we simulate a low data scenario and a high data scenario. Our dataset, tasks, models, evaluation metrics, and experimental setup are described in greater detail in the following sections.

#### Dataset, tasks, and pre-processing

Our dataset consists of cancer pathology reports obtained from the Louisiana Tumor Registry (LTR), Kentucky Cancer Registry (KCR), Utah Cancer Registry (UCR), and New Jersey State Cancer Registry (NJSCR) of the SEER Program.^2^ The study was executed in accordance to the institutional review board protocol DOE000152. Each pathology report in our dataset is associated with a unique tumor ID; the same tumor ID may be associated with one or more pathology reports. For each tumor ID, one or more human CTRs manually assigned ground truth labels for key data elements such as cancer site and histology based on all data available for that tumor ID. We note that these ground truth labels are at the tumor level rather than at the report level; as a consequence of this labelling scheme, tumor IDs associated with multiple pathology reports may have a tumor-level label that does not reflect the content within individual pathology reports. Thus, for this study, we only utilize tumor IDs associated with a single pathology report. The resulting LTR, KCR, UCR, and NJSCR datasets consist of 61,123, 46,859, 21,705, and 70,665 pathology reports respectively, yielding a total of 200,352 documents for our experiment.

For this study, we focus on identifying two key data elements that are of importance to NCI—subsite, which is used to identify cancer topology and is indicated by a 3-digit code, and histology, which is used to identify cancer morphology and is indicated by a 4-digit code. Furthermore, these two tasks were chosen because they have a very large number of possible classes and thus are especially challenging for automated machine learning methods. In our dataset, each pathology report is labelled with one of 317 possible subsites and one of 525 possible histologies. For a full list of possible subsite and histology labels and their details, we refer readers to the official SEER program coding and staging manual.^3^ The two figures in Additional file 1 show the number of occurrences per label of the 50 most frequent classes for histology and subsite. We can see from the figures that there is extreme class imbalance—some classes are represented by less than a few hundred pathology reports, while others are represented by tens of thousands of reports.

**Table 1:** Data split and number of classes for the two tasks analyzed

Dataset	Initial training	Validation	Testing	Holdout	Classes	
Large	15,000	18,032	20,036	147,284	525 (Histology)	317 (Subsite)
Small	1000	18,032	20,036	161,284	525 (Histology)	317 (Subsite)

Similar to our previous studies, we applied standard text pre-processing techniques such as lowercasing and tokenization to clean our corpus [1, 2]; these steps are described in detail in Additional file 2. After pre-processing, the average pathology report is 610 word tokens. To reduce the vocabulary size, all words with document frequency less than five were replaced with an “$unknown\_word$” token, all decimals were converted to a “*decimal*” word token, and all integers larger than 100 were converted to a “$large\_integer$” word token. We limit the maximum length for each cancer pathology report to 1500 word tokens; reports longer than 1500 tokens are truncated and reports shorter than 1500 tokens are zero-padded.

For our active learning setup, we require four data splits: (1) an initial annotated train set to train the starting model used for active learning, (2) an annotated validation set to use for early stopping to prevent overfitting, (3) an annotated test set for performance evaluation, and (4) an unannotated holdout set on which active learning is applied to select new entries to annotate with ground truth labels and add to the train set. Using our cleaned corpus, we create two datasets to simulate active learning situations with different amounts of labelled data. For our first dataset, we begin with a labelled training set of 15K samples; this dataset represents an active learning scenario with a fairly large amount of labelled data. For our second dataset, we begin with only 1K labelled reports for the initial training set; this dataset represents an active learning scenario with a small amount of labelled data. We use the exact same validation and test sets for both scenarios, and the holdout set is comprised of all remaining samples. Table 1 shows the size of each dataset partition that was used to simulate each active learning scenario.

#### TextCNN

For our classification model, we use a word-level text CNN because it is widely used for clinical NLP and text classification tasks [3, 29]. The CNN architecture is implemented based on the same architecture we have used in previous studies [1, 2]. The model hyperparameters are listed in Table 2. Our CNN uses randomly initialized word embeddings, which perform as well as or better than other pre-trained word embeddings when applied to our particular dataset, model, and experimental setup [30]. When training our CNN, we checkpoint after each epoch and stop training if validation accuracy does not improve for five consecutive epochs; we test using the checkpoint from the epoch with the best validation accuracy.

**Table 2:** CNN hyperparameters

Input length	Word embed dim	Num filters	Conv window sizes	Dropout	Optimizer	Learning rate	Batch size
1500	300	100	3, 4, and 5	0.5	Adam	0.0001	128

#### Evaluation metrics: F1 score

To evaluate the performance of each active learning method, we calculate the micro F1 score (Eq. 11) of the CNN after each iteration of active learning. We note that micro F1 score is equivalent to classification accuracy in classification tasks such as ours in which each sample is assigned to exactly one class. Micro F1 score is an important metric because it reflects the overall percentage of reports classified correctly.

Because micro F1 score measures overall accuracy regardless of class, in classification tasks with extreme class imbalance, the micro F1 score mostly reflects the performance on majority classes. Therefore, we also report the macro F1 score (Eq. 12) of the CNN after each iteration. Macro F1 score equally weighs the F1 score on each unique class regardless of class size. As a result, macro F1 score is more heavily influenced by performance on minority classes. In our application, it is important that automated classifiers correctly identify cancer subsites and histologies even if they are rare; as such, macro F1 score is an useful indicator of the effectiveness of each active learning method on the rare classes.

9 $$\begin{aligned}&\text {Precision} = \frac{\textit{True Positive}}{\textit{True Positives} + \textit{False Positives}} \end{aligned}$$

10 $$\begin{aligned}&\text {Recall} = \frac{\textit{True Positives}}{\textit{True Positives} + \textit{False Negatives}} \end{aligned}$$

11 $$\begin{aligned}&\text {Micro F1} = 2*\frac{\textit{Precision * Recall}}{\textit{Precision} + \textit{Recall}} \end{aligned}$$

12 $$\begin{aligned}&\text {Macro F1} = \frac{1}{|C|} \sum _{C_i}^{C} F1(C_i) \end{aligned}$$

In Eq. 12, $C_i$ represents the subset of training samples belonging to class *i*, and |*C*| is the total number of possible classes.

#### Evaluation metrics: class imbalance

Extreme class imbalance is a common and problematic issue in clinical classification tasks, and it is often difficult to effectively train a classifier on a class with very few labelled samples. To better understand how active learning can reduce extreme class imbalance, we analyze the class imbalance of the training dataset after each iteration of active learning. We use a modified version of Shannon Entropy as a balance metric, described in Eq. 13:

13 $$\begin{aligned} \textit{Balance} = \frac{-\sum _{i=1}^{C}\frac{c_i}{n} * \log (\frac{c_i}{n})}{\log C} \end{aligned}$$

where *n* represents the number of documents in the training set, *C* is the total number of possible classes (525 for histology and 317 for subsite), and $c_i$ is the number of training samples that belong to class *i*. This equation outputs a value of 0 when the training dataset is perfectly imbalanced (i.e., contains only samples from a single class) and a value of 1 when the training dataset is perfectly balanced (i.e., all classes have the same number of training samples).

#### Evaluation metrics: proportion of unique classes

We also track how active learning affects the number of unique classes seen by the model after each iteration. Intuitively, a model that has never seen samples from a rare class will never accurately predict that class; therefore we want to expose the model to as many unique classes as possible. To measure this, after each iteration of active learning, we simply calculate the ratio between the number of classes present in the training dataset and the total number of classes within the entire dataset.

#### Experimental setup

To compare the performance of the different active learning strategies, we benchmark using two different datasets simulating low and high resource settings (see Table 1). For each of these two datasets, we test the effectiveness of the CNN models on two different tasks—subsite and histology. These are treated as two independent single-task classification problems—we train one CNN to predict subsite and a separate CNN to predict histology. This results in a total of four different active learning experiments.

**Fig. 1:**
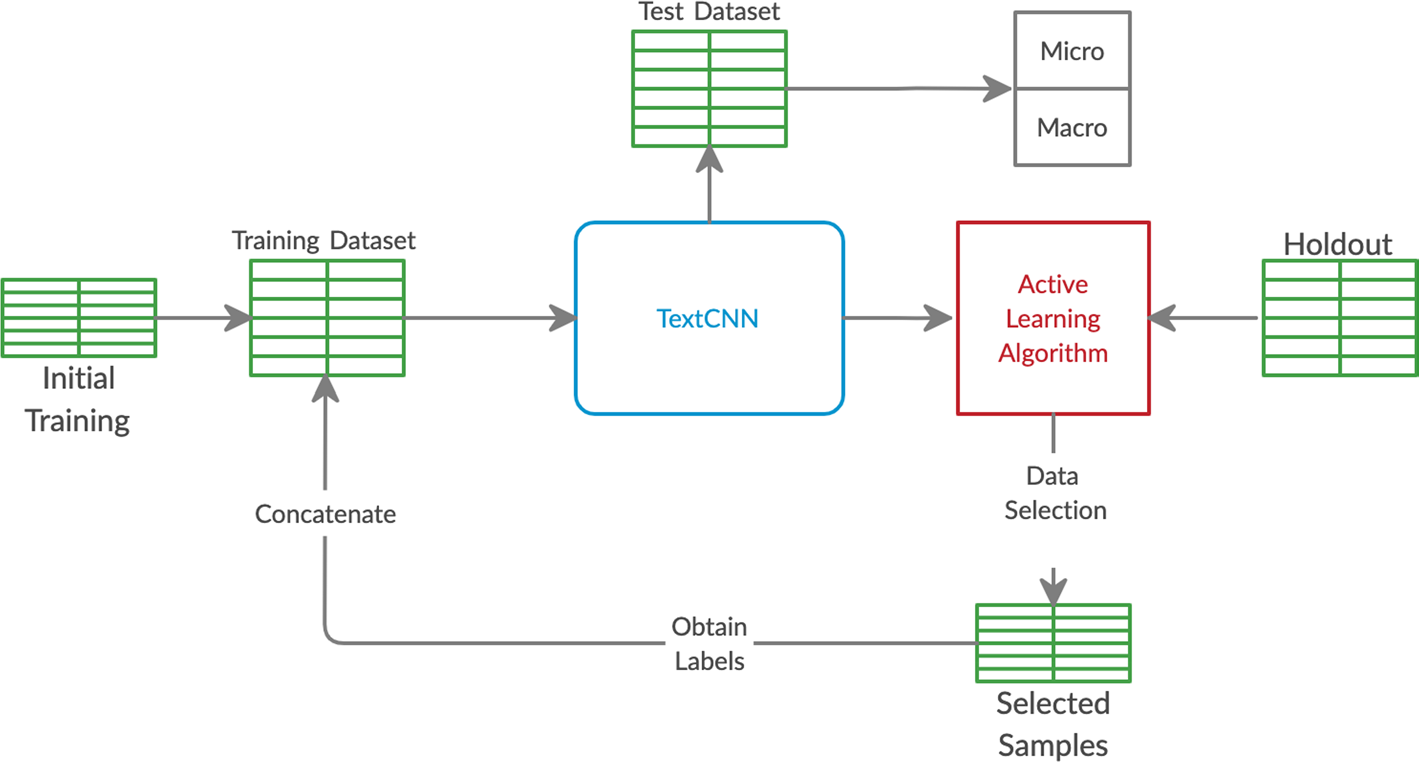
Flowchart of the computational pipeline used during the active learning experiments

For any given active learning strategy, we first train a CNN on the initial training set. Then, we use that active learning strategy with the trained CNN to select a subset of reports from the holdout set with the highest $\phi$; we select 1K samples per active learning iteration for the small dataset and 15K samples per active learning iteration for the large dataset. These selected reports are removed from the holdout set and added to the training set along with their ground truth labels, and a new CNN is trained from scratch on the new training set. For the large dataset, this process is repeated until there are no remaining documents in the holdout set. For the small dataset, we repeat this process nine times total until the training set consists of 10K samples. Figure 1 shows a general flowchart of the computational pipeline followed during each experiment.

**Fig. 2:**
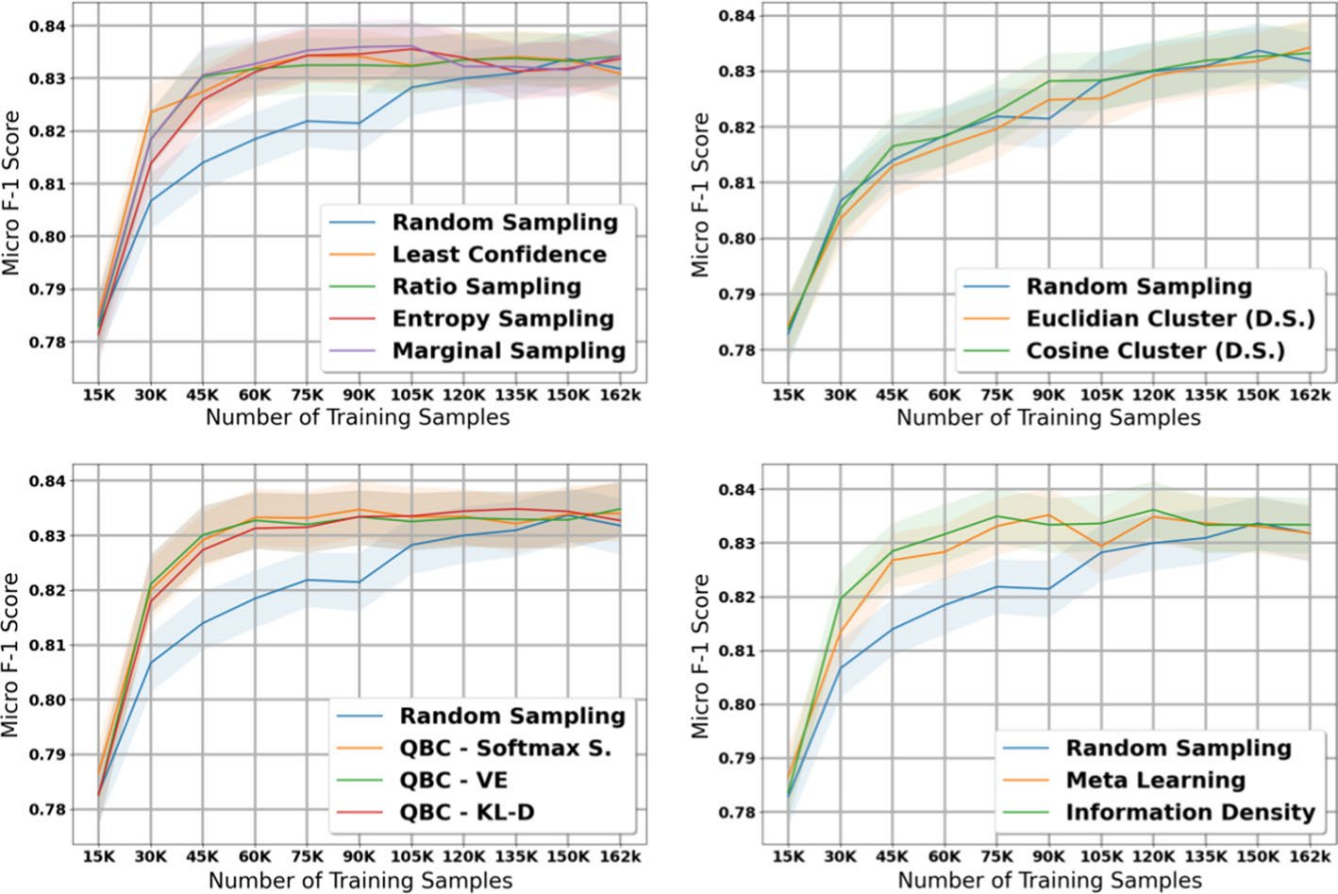
Micro score results for the 11 active learning algorithms applied during the large dataset experiment on histology. Blue line represents random sampling

At each iteration of active learning, we report the micro and macro F1 scores on the test set, the class imbalance of the training set, and the proportion of unique classes seen by the model. For micro F1 score, we calculate 95% confidence intervals using a bootstrapping procedure [31] described in detail in Additional file 3. We note that we do not use bootstrapping on the macro F1 score because it tends to undersample the minority classes, which are critical for accurately representing macro F1 score. Also, we note that for all active learning strategies and at every iteration of active learning, the test and validation sets are fixed to maintain consistency.

After each iteration of active learning, we train a new CNN from scratch (i.e., cold start) rather than continue training the weights from the previous CNN (i.e. warm start); this is because we found that warm start results in lower accuracy, especially in the later iterations of active learning. We provide a comparison plot between active learning with cold start, active learning with warm start, and no active learning from one of our experiments in Additional file 4.

All experiments are run using Tensorflow 1.15 and a single NVIDIA V100 GPU (we note that the QBC models are trained in parallel, with each committee member trained on a single NVIDIA V100 GPU). For reference, we report the training and inference time for one of our experiments using the large dataset in Additional file 5. We note that the text CNN model that we use is a relatively simple DL model with approximately 22M learnable parameters, 20M of which are associated with the learnable word embeddings. Using our experimental settings (see Table 2), the model will train even on lower-end GPUs with less than 4 GB memory.

## Results

### Histology—Large dataset

**Fig. 3:**
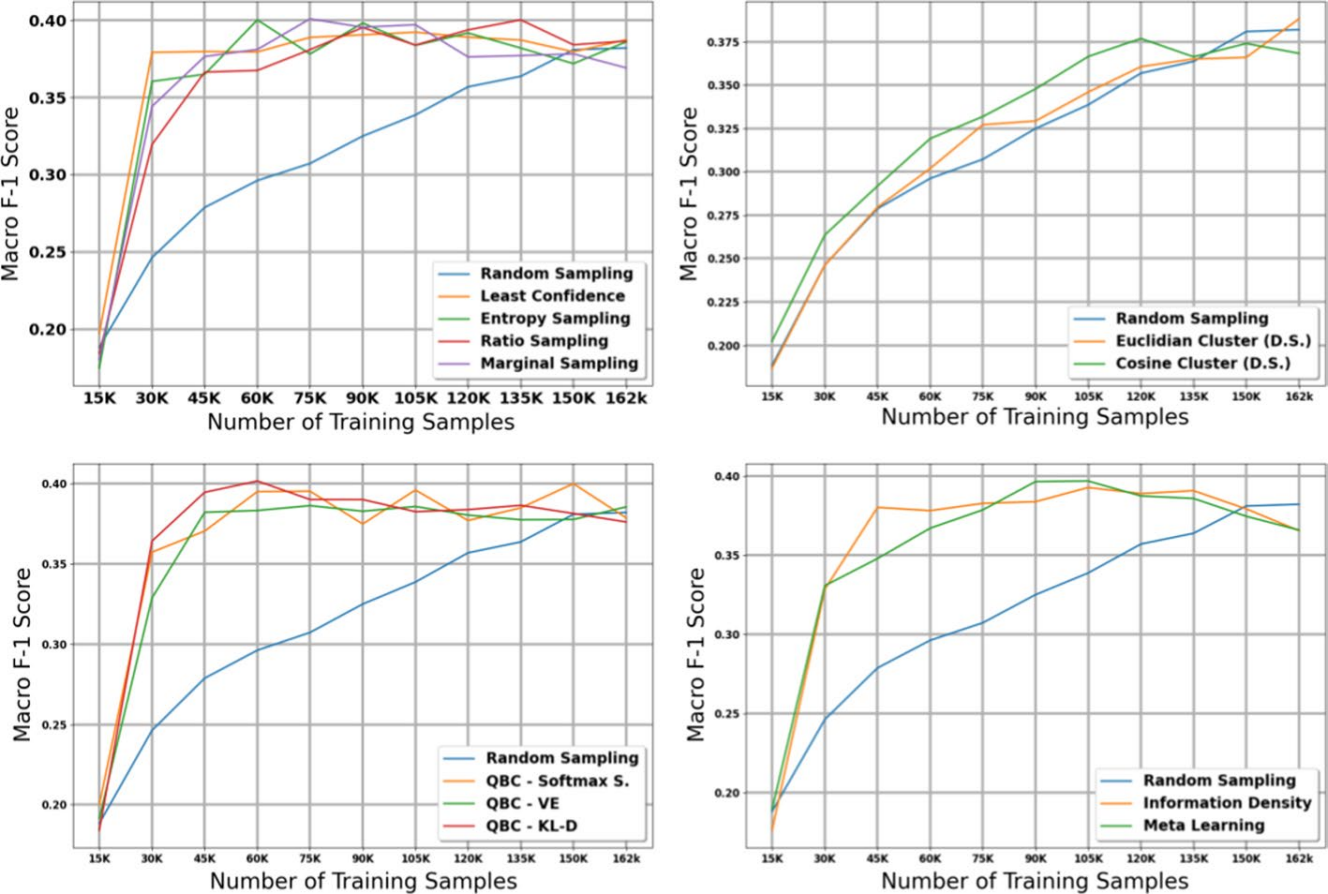
Macro score results for the 11 active learning algorithms applied during the large dataset experiment on histology. Blue line represents random sampling

The table in Additional file 6 shows the micro and macro F1 scores for the histology task using our large dataset with 15K initial training samples; we also plot the results with shaded 95% confidence intervals (Figs. 2, 3). After accounting for the confidence intervals, all active learning strategies implemented in this paper except for the diversity-based methods performed significantly better than the baseline of no active learning, i.e., random sampling. We note that this difference in performance between active learning and random sampling decreases in the later iterations; this is expected because by the last iteration of active learning, all methods (including random sampling) are training on the exact same data, i.e., all data available in the holdout set.

Diversity sampling, which makes decisions based on the similarity between document embeddings created by the CNN, did not produce significant improvements over random sampling. These results suggest that the document embeddings generated by the CNN, which are optimized for classification, may not adequately capture the information necessary to distinguish informative documents. Furthermore, euclidean and cosine distance from the nearest class centroid may not be the best indicator of how informative a document is.

After excluding the diversity sampling strategies, no single active learning strategy stands out as a clear winner in terms of micro F1 scores after accounting for confidence intervals. In terms of macro F1 scores, QBC with KL divergence obtained the strongest macro scores in early iterations, but most of the active learning methods managed to reach the maximum macro F1 score of $\sim$0.40.

One interesting observation is that the highest macro scores tend to appear towards the middle iterations of the experiment and then tend to go down during the last few iterations. This is a pattern that is not observed for micro scores, where the maximum score is attained near the middle iterations and remains high throughout the rest of the iterations. We expect that this is because in the later iterations, most of the data remaining in the holdout set are less informative samples from majority classes; adding these samples does not negatively affect overall accuracy but may increase class imbalance and thus reduce performance on minority classes.

### Subsite—Large dataset

**Fig. 4:**
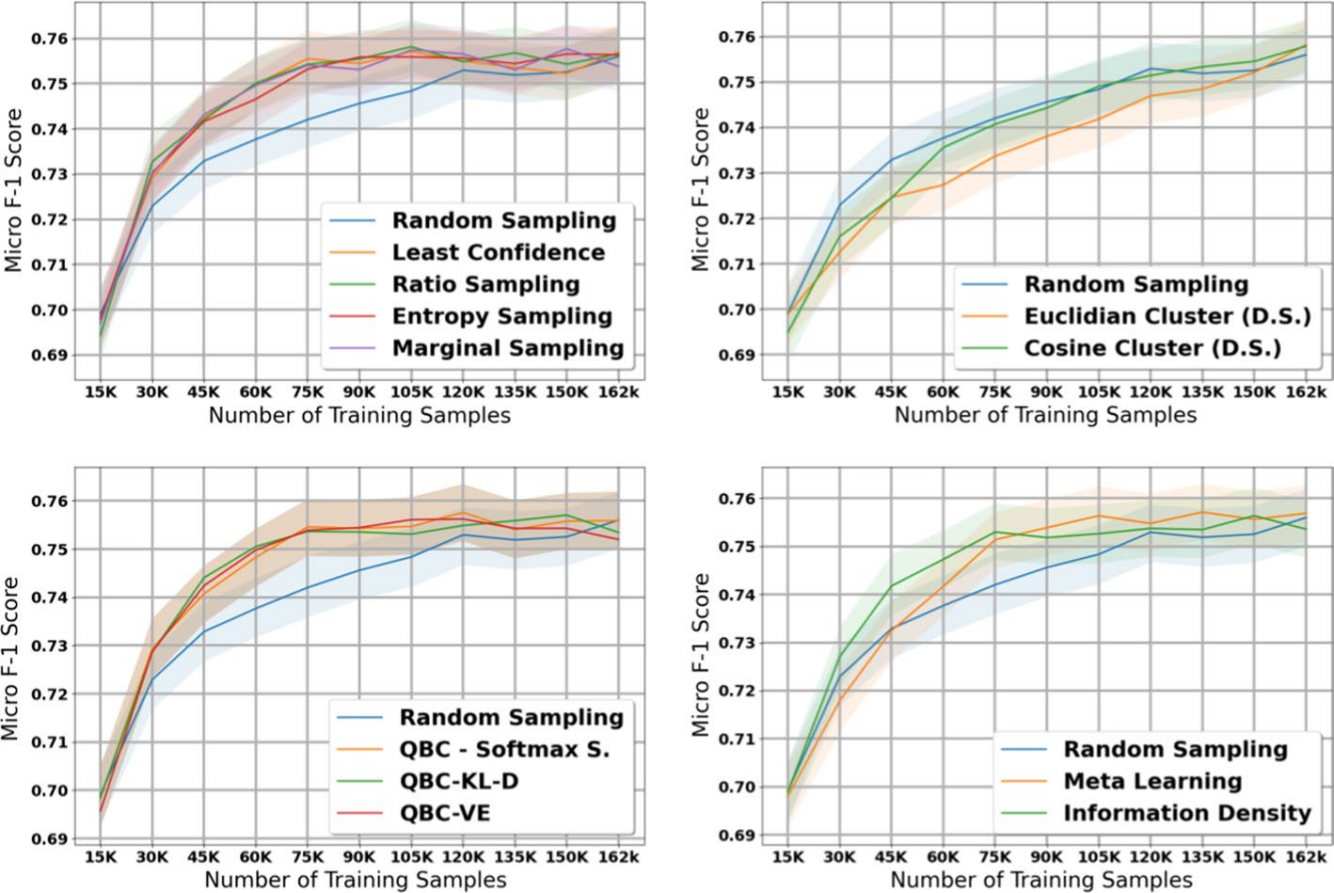
Micro score results for the 11 active learning algorithms applied during the large dataset experiment on subsite. Blue line represents random sampling

**Fig. 5:**
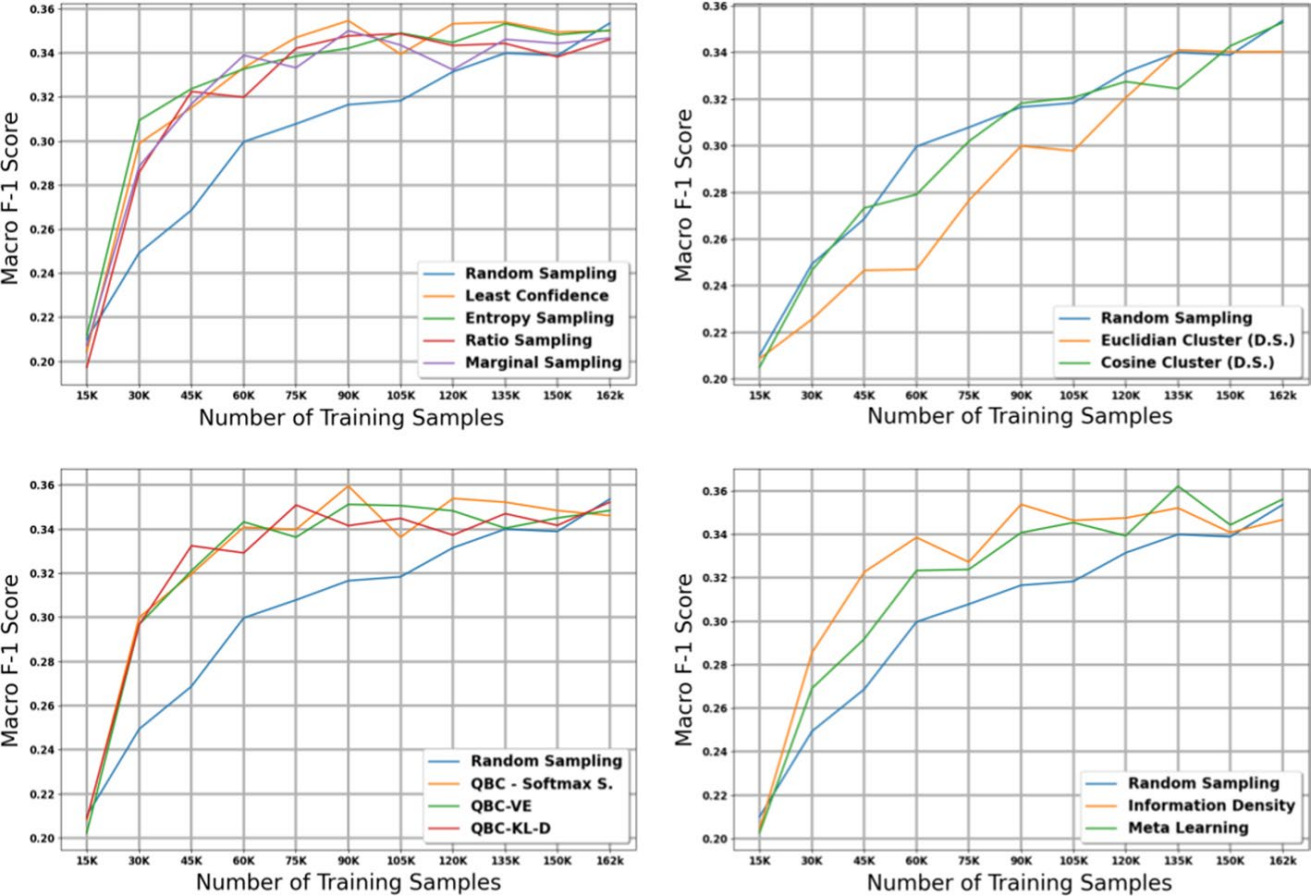
Macro score results for the 11 active learning algorithms applied during the large dataset experiment on subsite. Blue line represents random sampling

The table in Additional file 7 shows the micro and macro F1 scores for the subsite task using our large dataset with 15K initial training samples; Figs. 4 and 5 provide plots of our results with 95% confidence intervals. The results on our subsite task were very similar to our results from our histology task. After accounting for confidence intervals, the diversty-based methods failed to perform significantly better than random sampling, and Euclidian distance actually performed worse than random sampling. All other methods attained maximum micro and macro F1 scores much earlier than random sampling.

Once again, after excluding the diversity-based strategies, it is difficult to distinguish a clear winner in terms of micro F1 score after accounting for confidence intervals. The weakest method appears to be Meta Learning, which performed similarly to random sampling in the first three iterations of the experiment. In terms of macro F1 score, the QBC methods once again attained strong macro scores at early iterations of the experiment; however, most of the other methods manage to reach the maximum macro F1 score of $\sim$0.35 by the middle iterations of the experiment.

We note that in the subsite experiment, we do not observe the same drop in macro F1 scores toward the later iterations of active learning that we observed in the histology experiment. We expect that this is because there are fewer unique classes in the subsite task compared to the histology task, and thus the effect of class imbalance is less severe.

### Histology—Small dataset

**Fig. 6:**
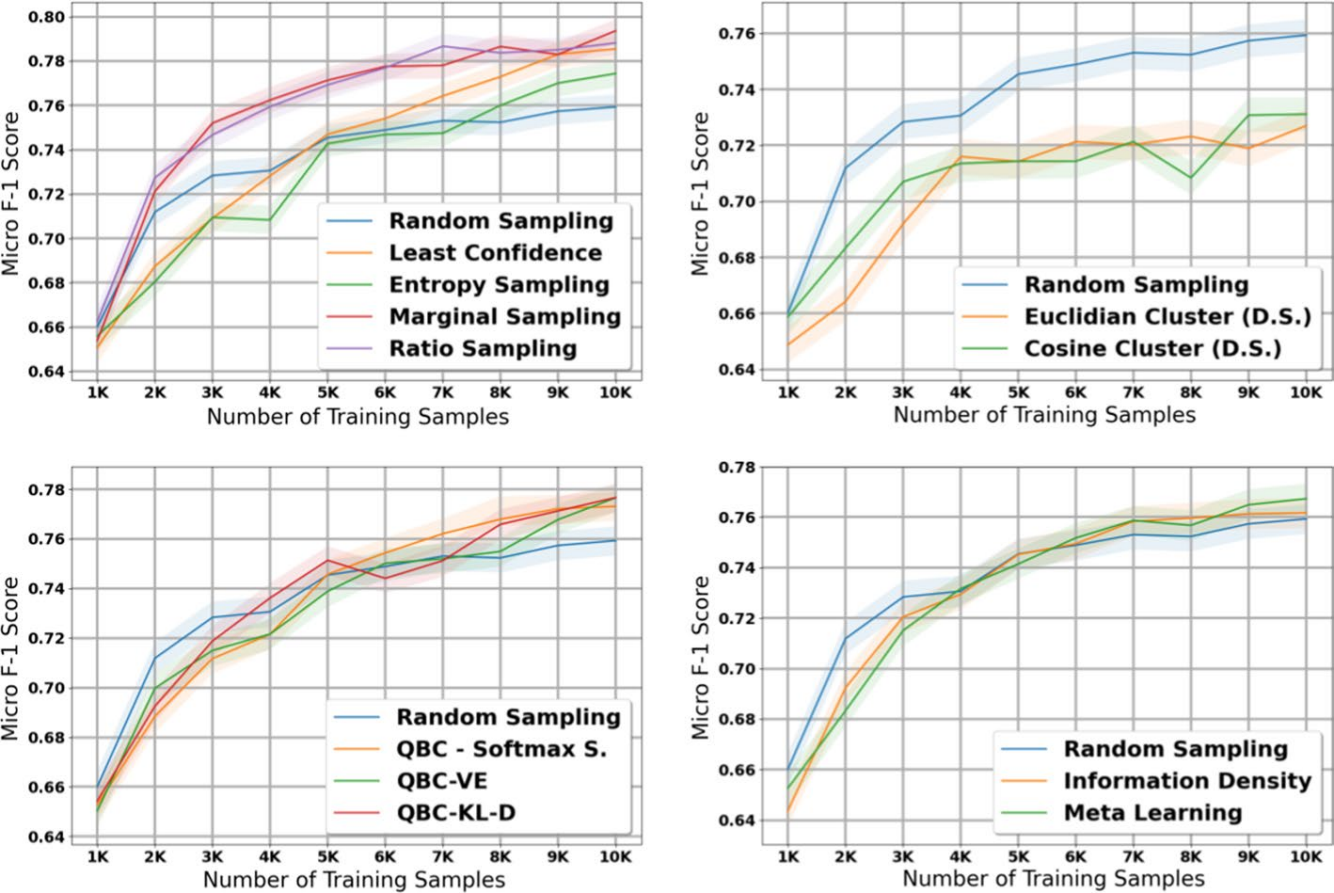
Micro score results for the 11 active learning algorithms applied during the small dataset experiment on histology. Blue line represents random sampling

**Fig. 7:**
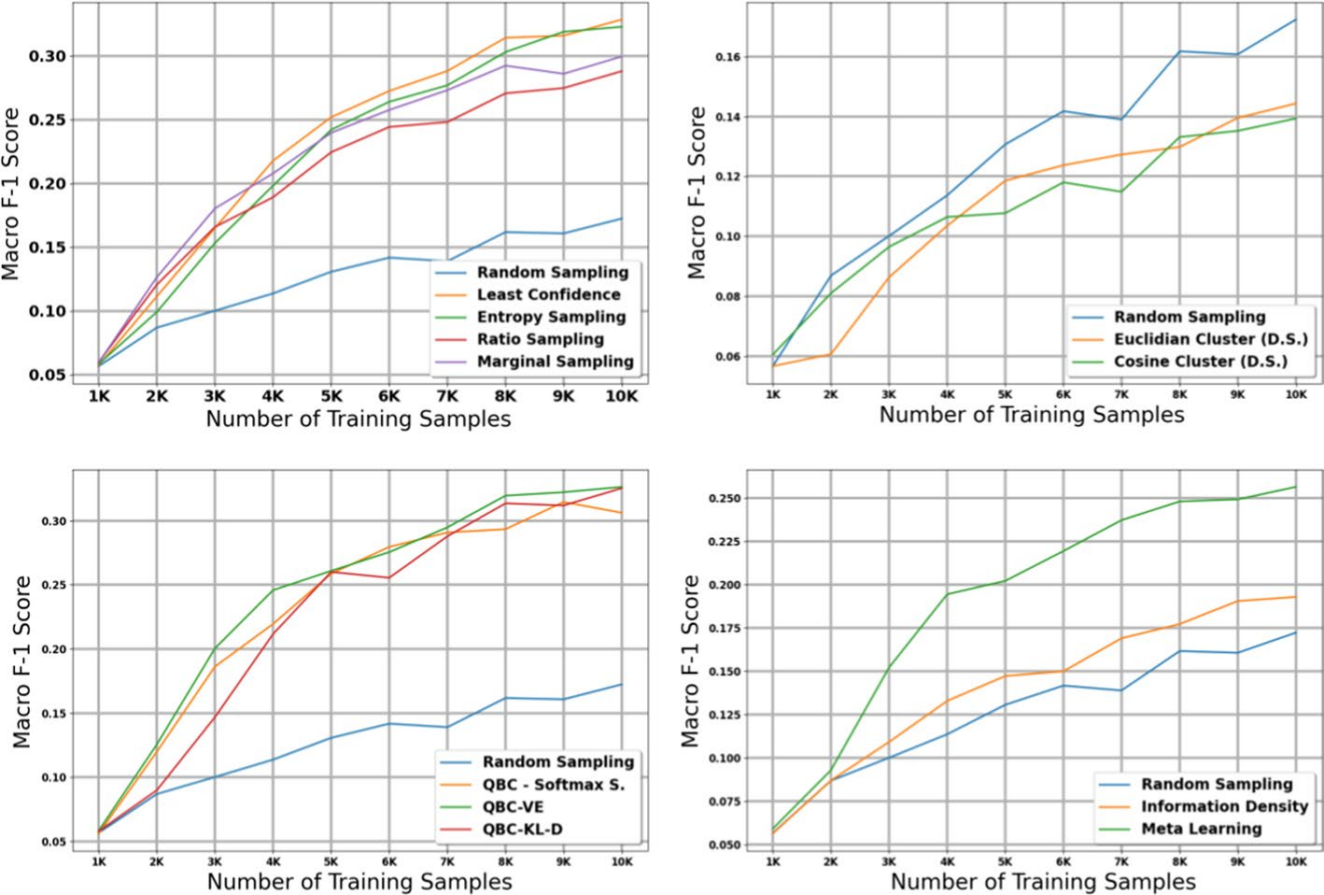
Macro score results for the 11 active learning algorithms applied during the small dataset experiment on histology. Blue line represents random sampling

The table in Additional file 8 shows the micro and macro F1 scores for the histology task using our small dataset with 1K initial training samples; we also plot the results with shaded 95% confidence intervals in Figs. 6 and 7. Compared to the experiments on the large dataset, we notice several important similarities and differences. First, the diversity sampling strategies not only failed to outperform random sampling, but performed significantly worse in these experiments. Secondly, most active learning strategies that had solid performance in the large dataset no longer show strong performance in this small dataset—the QBC strategies, least confidence, entropy sampling, information density, and meta learning all failed to perform better than random sampling in most of the early and middle iterations.

After taking into account confidence intervals, the marginal sampling and ratio sampling techniques were the only two active learning techniques that significantly outperformed the random sampling baseline in terms of micro F1 score. Interestingly, these two methods do not attain the best performance in macro F1 score, class balance, and proportion of unique classes seen by the model.

When examining macro F1 score, all active learning strategies except for the diversity sampling strategies and information density perform much stronger than the random sampling baseline. These results suggest that in general, the active learning strategies in this paper focus on minority classes at the expense of the majority classes; in some cases this may increase macro F1 score but reduce the potential gains in micro F1 score. We explore this phenomena in greater detail in our Discussion.

### Subsite—Small dataset

**Fig. 8:**
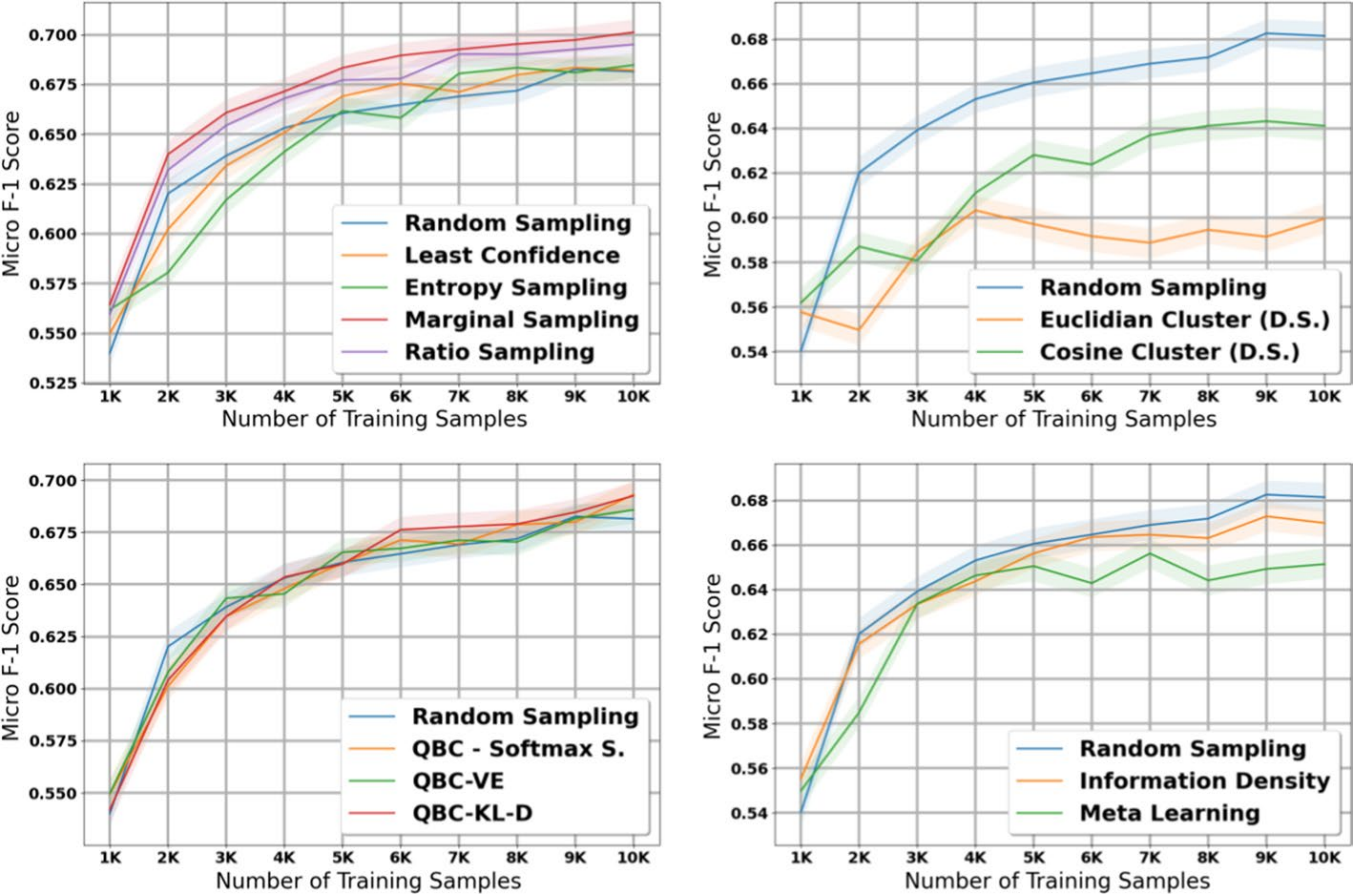
Micro score results for the 11 active learning algorithms applied during the small dataset experiment on subsite. Blue line represents random sampling

**Fig. 9:**
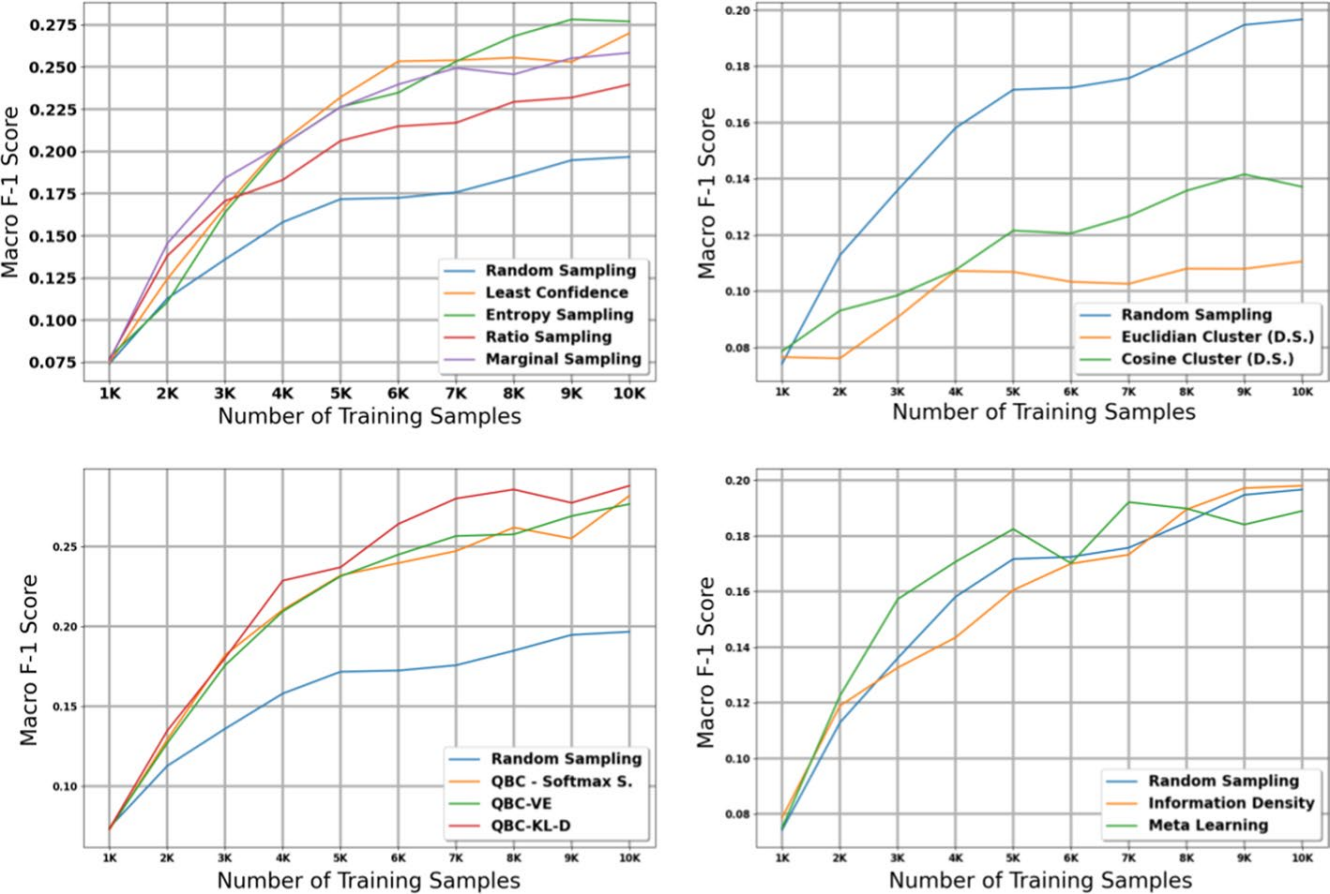
Macro score results for the 11 active learning algorithms applied during the small dataset experiment on subsite. Blue line represents random sampling

The table in Additional file 9 shows the micro and macro F1 scores for the subsite task using our small dataset with 10K samples; we also plot the results with shaded 95% confidence intervals in Figs. 8 and 9. The findings in this experiment are similar to our findings in the histology experiment with the small dataset.

In terms of micro F1 score, marginal sampling and ratio sampling are the only two active learning strategies that significantly outperform the baseline of random sampling, and meta learning and the diversity sampling strategies significantly underperformed random sampling. However, in terms of macro F1 score, all methods except for the diversity sampling strategies outperform the random sampling baseline. Combined with the histology results on our small dataset, these results suggest that the best active learning strategy depends on the size of the initial training set and the amount of labelled data added per iteration of active learning.

## Discussion

Our experiments show that the best choice of active learning strategy is dependent on the size of the initial labelled training set and the amount of labelled data added per iteration. In our large dataset experiments, there was no clear winner in terms of micro F1 score (overall accuracy)—least confidence, ratio sampling, entropy sampling, marginal sampling, QBC softmax, QBC VE, QBC KL-D, and information density all had similar performance. If macro F1 score and performance on minority classes is of high importance, QBC KL-D had the strongest performance on early iterations; however this difference is not huge and the other high-performing active learning strategies achieve similar macro F1 score in later iterations. Therefore, the choice of best active learning strategy may simply come down to choosing the most computationally efficient method that is fastest to run.

From the algorithms implemented in this paper, the QBC techniques are the most computationally expensive because each iteration of active learning requires training a full committee of models. Other techniques involving document embeddings and clustering techniques (information density and diversity sampling) are also compuationally expensive because they involve repetitive computation of distance between large vectors. The most computationally inexpensive active learning approaches are the uncertainty sampling strategies because they require the least additional computation beyond what is already provided by the base classification model. Consequently, these strategies are favored in settings with a high amount of initial labelled data and a large amount of labelled data added per iteration of active learning.

In our small dataset experiments, the marginal sampling and ratio sampling techniques obtained significantly better micro F1 scores than all other active learning techniques. However, these two methods did not maintain the best overall macro F1 scores; the best macro F1 scores were obtained by least confidence, entropy sampling, and the QBC approaches.

One possible explanation for this phenomenon is the impact of the majority classes on overall micro and macro F1 score. Both our histology and subsite tasks are characterized by extreme class imbalance. In the histology task, the 10 most common classes make up $\sim$60% of the dataset, and in the subsite task, the 10 most common classes make up $\sim$50% of the dataset; the class distributions are available in Additional file 1. Thus, performing well on the majority classes greatly impacts micro F1 score while having a slight effect on macro F1 score.

On the large dataset with 15k initial labelled samples, the base model observes hundreds or thousands of samples from the majority classes. After a few additional iterations of active learning, it is likely that the classifier achieves strong performance on the majority classes, and thus additional gains in overall micro F1 score must account for the minority classes. We see this trend across all the successful active learning strategies—the best performing strategies have strong performance in both micro and macro F1 score.

However, on the small dataset with 1K initial labelled samples, the classifier is unlikely to have strong performance on the majority classes (or any other classes) mainly because it has not seen a sufficient number of samples. Thus, active learning methods that focus on maximizing performance on the majority classes achieve better overall micro F1 score than strategies that focus on minority classes because the majority classes make up a larger portion of the test set; however, this may come at the expense of performance on the minority classes and therefore reduce macro F1 score. We observe this trend in our small dataset results—the two best performing strategies, marginal sampling and ratio sampling, do not have the best macro F1 scores after the initial two iterations of active learning.

**Fig. 10:**
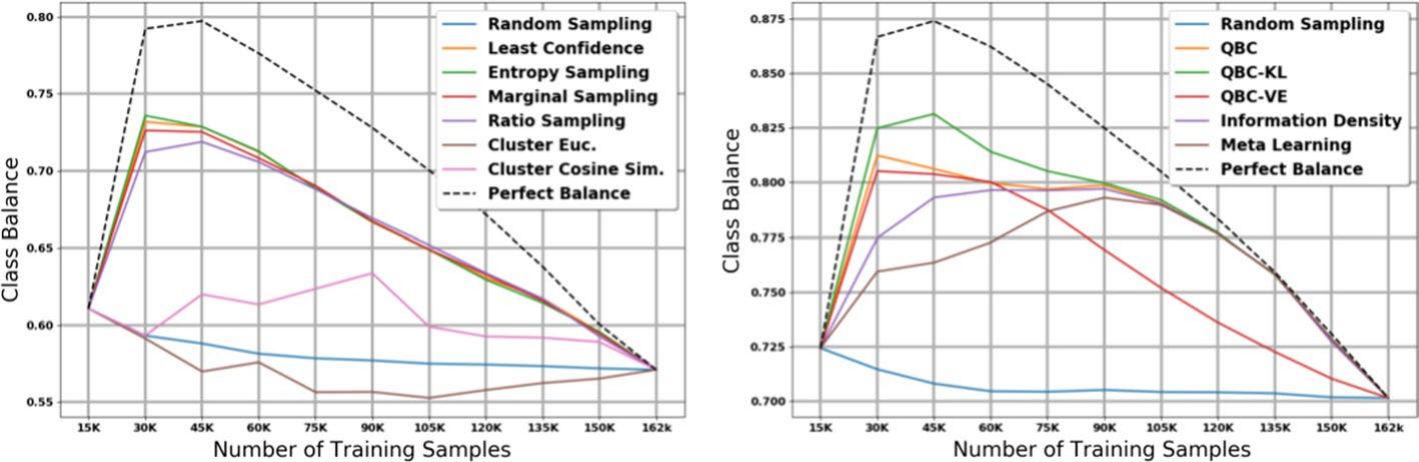
Class imbalance Plots. Black line represents the upper limit (most balance dataset possible). Y-values are computed with Eq. 13: $y=0$ represents no balance, and $y=1$ represents full balance

**Fig. 11:**
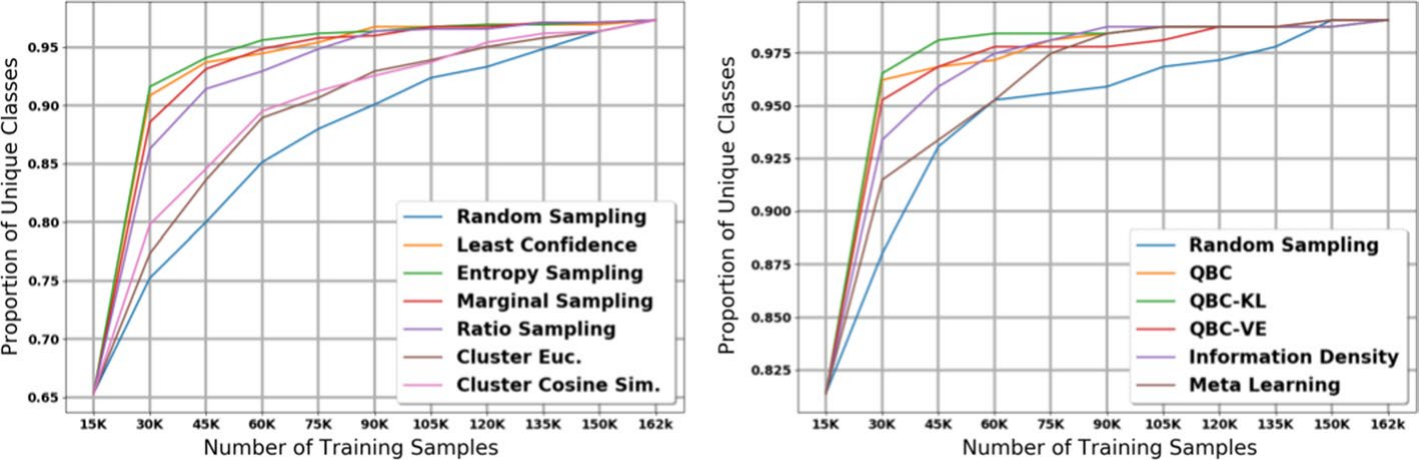
Proportion of classes seen by the models at each iteration. The *y* values consists of the number of unique classes present in the training dataset divided by the total number of classes in each task (525 for histology and 317 for subsite)

A large number of unique labels with extreme class imbalance is a common property of many clinical text applications such as ours. To better understand how active learning affects performance on minority classes, we plot the class imbalance within the training dataset (Fig. 10, Additional files 10, 11, 12, and 13) and the number of unique classes seen in the training dataset (Fig. 11, Additional files 14, 15, 16, and 17) after each iteration of active learning. Not surprisingly, for any given active learning strategy, there is a direct correlation between the macro F1 scores, the class balance, and the number of unique classes seen by the model.

Furthermore, we also examine how active learning affects the distribution of the training data compared to random sampling by visualizing the document embeddings in the final training set for the small histology experiment after 10 iterations of ratio sampling and 10 iterations of random sampling (Additional file 18). Documents embeddings are extracted from the penultimate layer of the CNN and reduced to 2D via t-distributed stochastic neighbor embedding (TSNE), and we color each document embedding based off whether it belongs to a majority class (number of total samples in dataset above average) or minority class (number of total samples in dataset below average). After 10 iterations of random sampling, 89.4% of documents in the training set belonged to majority classes and 10.6% belonged to the minority classes, while after 10 iterations of ratio sampling 72.1% of documents in the training set belonged to majority classes and 27.9% belonged to minority classes. Compared to random sampling, ratio sampling increases the overall percentage of minority classes in the training set; our visualization shows that many of these minority class documents form new small, well-defined clusters or expand the size of other small, existing clusters. We hypothesize that these documents play a large role in improving macro F1 score. We also note that some of the documents from the minority classes end up in the center without any clear clustering. This is likely because active learning may choose samples from extremely rare classes that do not yet cluster due to lack of training data or ambiguous edge cases that are difficult to classify; these samples may negatively affect overall micro F1 score in our small dataset experiments.

Our analysis of class balance and unique labels supports our hypothesis that in high data availability environments with a large amount of initial labelled data, boosting performance on minority classes is important for micro F1 score. On the other hand, in low data availability environments with a small amount of initial labelled data, it is more important to focus on majority classes to improve micro F1 score. In the early iterations of our large dataset experiments, we see that the most effective active learning strategies (uncertainty sampling and QBC) also generate the highest class balance and unique classes in the training set. On the other hand, the two best active learning strategies in our small data experiments—marginal and ratio sampling—have lower class balance and unique classes compared to least confidence, entropy sampling, and the QBC strategies. Overall, this analysis suggests that in applications with a high number of unique labels and extreme class imbalance, active learning can play an important role in mitigating class imbalance such that rare classes have better representation in a given labelled dataset.

**Table 3:** Summary of effectiveness of each active learning strategy in across different key characteristics

	Overall micro (Large)	Overall macro (Large)	Overall micro (Small)	Overall macro (Small)	Single iteration micro (Small)	Single iteration macro (Small)	Compute cost
Least Con.	High	High	Med	High	Low	Med	Low
Entropy Sam.	High	High	Med	High	Low	Med	Low
Ratio Sam.	High	High	High	Med	High	High	Low
Marginal Sam.	High	High	High	Med	High	High	Low
Euclidian C. (D.S.)	V. Low	V. Low	V. Low	V. Low	V. Low	V. Low	Med
Cosine C. (D.S.)	V. Low	V. Low	V. Low	V. Low	V. Low	V. Low	Med
Information Den.	High	High	Low	Low	Low	Low	Med
Meta Learning	Med	Med	Low	Low	Low	Low	Med
QBC—S.S.	High	High	Med	High	Low	High	High
QBC—VE	High	High	Med	High	Low	High	High
QBC—KL-D	High	High	Med	High	Low	High	High

As mentioned in our results, Fig. 3 and Additional file 6 show that macro F1 scores dropped in the later iterations of the large dataset histology experiments. The balance and unique class proportion plots explain this unintuitive phenomenon. The overall class balance of the training dataset is much higher in the early iterations than in the later iterations. This is because by the later iterations, there are few or no samples from the minority classes left in the holdout set; consequently, the dominant classes and the least informative documents start to fill out the training dataset. As we have seen, lower class balance also correlates with lower macro F1 scores. Thus, in the later iterations, while active learning achieves high micro F1 scores, the performance on uncommon classes decreases because they make up a much smaller portion of the training set.

In Table 3, we summarize our findings regarding the effectiveness of each active learning strategy across three key characteristics—(1) overall performance in terms of micro and macro F1 scores, (2) effectiveness after only a single iteration of active learning with 1K additional labelled samples, which may be important in low resource settings where additional labels are difficult or expensive to obtain, and (3) computational cost to implement the strategy. Based on this table, we conclude that the ratio sampling and marginal sampling strategies are strong contenders for best overall active learning strategy because they have overall strong performance in both the high data availability and low data availability settings, have the best performance when additional labelled data is extremely limited, and are computationally very simple.

## Conclusions

In this work, we evaluated the effectiveness of 11 different active learning strategies in the context of classifying cancer subsite and histology from cancer pathology reports. Our dataset is characterized by a large number of unique labels with extreme class imbalance, and we use a text CNN as the base classification model. For each of our two classification tasks, we tested under two different active learning scenarios—(1) a high data availability setting where we start with 15K labelled samples and added an additional 15K labelled samples after each iteration of active learning, and (2) a low data availability setting where we start with 1K labelled samples and added an additional 1K labelled samples after each iteration of active learning. After each iteration of active learning, we reported the micro and macro F1 scores of the classifier as well as the class balance and unique labels in the training dataset.

We showed that in the high data availability setting, the uncertainty sampling and QBC strategies obtained the best overall micro F1 scores, and the QBC KL-D strategy obtained the best overall macro F1 score. In terms of micro F1 score, there was no single clear winner. In the low data availability setting, ratio and marginal sampling achieved the strongest overall micro F1 scores but underperformed slightly in macro F1 scores; least confidence, entropy sampling, and the QBC strategies obtained the best macro F1 scores. Ratio and marginal sampling are strong contenders for the overall best active learning strategy based on overall performance in the high and low data availability settings, performance when additional labelled data is extremely limited, and low computation cost. Compared to a model trained on all available data, active learning can obtain similar performance using less than half the data. Furthermore, on tasks with a large number of unique labels with extreme class imbalance, active learning can significantly mitigate the effects of class imbalance and improve performance on the rare classes.

The code for our text CNN and active learning algorithms will be released online prior to final publication.

## Supplementary Information

**Additional file 1.** Dataset class imbalance plots.

**Additional file 2.** Text preprocessing steps.

**Additional file 3.** Bootstrapping procedure for confidence.

**Additional file 4.** Performance of cold start ratio sampling, warm start ratio sampling, and random sampling on the histology task (small dataset).

**Additional file 5.** Training and inference time for the histology experiment (large dataset) using ratio sampling.

**Additional file 6.** Large dataset: micro/macro F-1 scores table - histology task.

**Additional file 7.** Large dataset: micro/macro F-1 scores table - subsite task.

**Additional file 8.** Small dataset: micro/macro F-1 scores table - histology task.

**Additional file 9.** Small dataset: micro/macro F-1 scores table - subsite task.

**Additional file 10.** Large dataset: class imbalance - histology task.

**Additional file 11.** Large dataset: class imbalance - subsite task.

**Additional file 12.** Small dataset: class imbalance - histology task.

**Additional file 13.** Small dataset: class imbalance - subsite task.

**Additional file 14.** Large dataset: class proportion plots - histology.

**Additional file 15.** Large dataset: class proportion plots - subsite task.

**Additional file 16.** Small dataset: class proportion plots - histology task.

**Additional file 17.** Small dataset: class proportion plots - subsite task.

**Additional file 18.** Document embeddings generated via TSNE for histology task (small dataset) with and without10 iterations of active learning. Documents are colored by majority class (number of total samples in dataset aboveaverage) and minority class (number of total samples in dataset below average).

## Data Availability

The data used for our experiments consists of cancer pathology reports that potentially contain identifiers as defined under HIPAA and would be protected health information; as such we are not authorized to make our dataset publicly available. The data that we have been provided has been done so under an approved IRB protocol and data use agreement with the data owners—the National Cancer Institute’s Louisiana, Kentucky, Utah, and New Jersey Surveillance, Epidemiology, and End Results Program (SEER) cancer registries. The data used in our experiments may be accessible, upon request and with subsequent authorized approvals, by individuals by contacting the Louisiana Tumor Registry (LTR-info@lsuhsc.edu), Kentucky Cancer Registry (ericd@kcr.uky.edu), Utah Cancer Registry (ucr.info@hsc.utah.edu), and New Jersey State Cancer Registry (webplushelp@cinj.rutgers.edu).

## Abbreviations

SEER: Surveillance, Epidemiology, and End Results
NCI: National Cancer Institute
NLP: Natural language processing
CNN/CNNs: Convolutional neural networks
DL: Deep learning
LC: Least confidence
MC: Marginal sampling
RC: Ratio confidence
ES: Entropy sampling
DS: Diversity sampling
EC: Euclidean cluster-based sampling
CC: Cosine cluster-based sampling
QBC: Query-by-committee
SS: Softmax sum
VE: Vote Entropy
KL: Kullback–Leibler
KL-D: Kullback–Leibler Divergence to the Mean
ID: Information Density
ML: Meta learning
LTR: Louisiana Tumor Registry
KCR: Kentucky Cancer Registry
UCR: Utah Cancer Registry
NJSCR: New Jersey State Cancer Registry
CTRs: Certified Tumor Registrars
TSNE: t-distributed stochastic neighbor embedding.

## Footnotes

1 https://www.cdc.gov/nchs/fastats/leading-causes-of-death.htm.

2 NJSCR is no longer in the SEER Program, but is included in the current data release.

3 https://seer.cancer.gov/tools/codingmanuals/index.html.
