# Supplementary figures and images for: Deep active learning for classifying cancer pathology reports
Source: BMC Bioinformatics. 2021 Mar 9;22:113. doi: 10.1186/s12859-021-04047-1 (PMC7941989; doi:10.1186/s12859-021-04047-1)

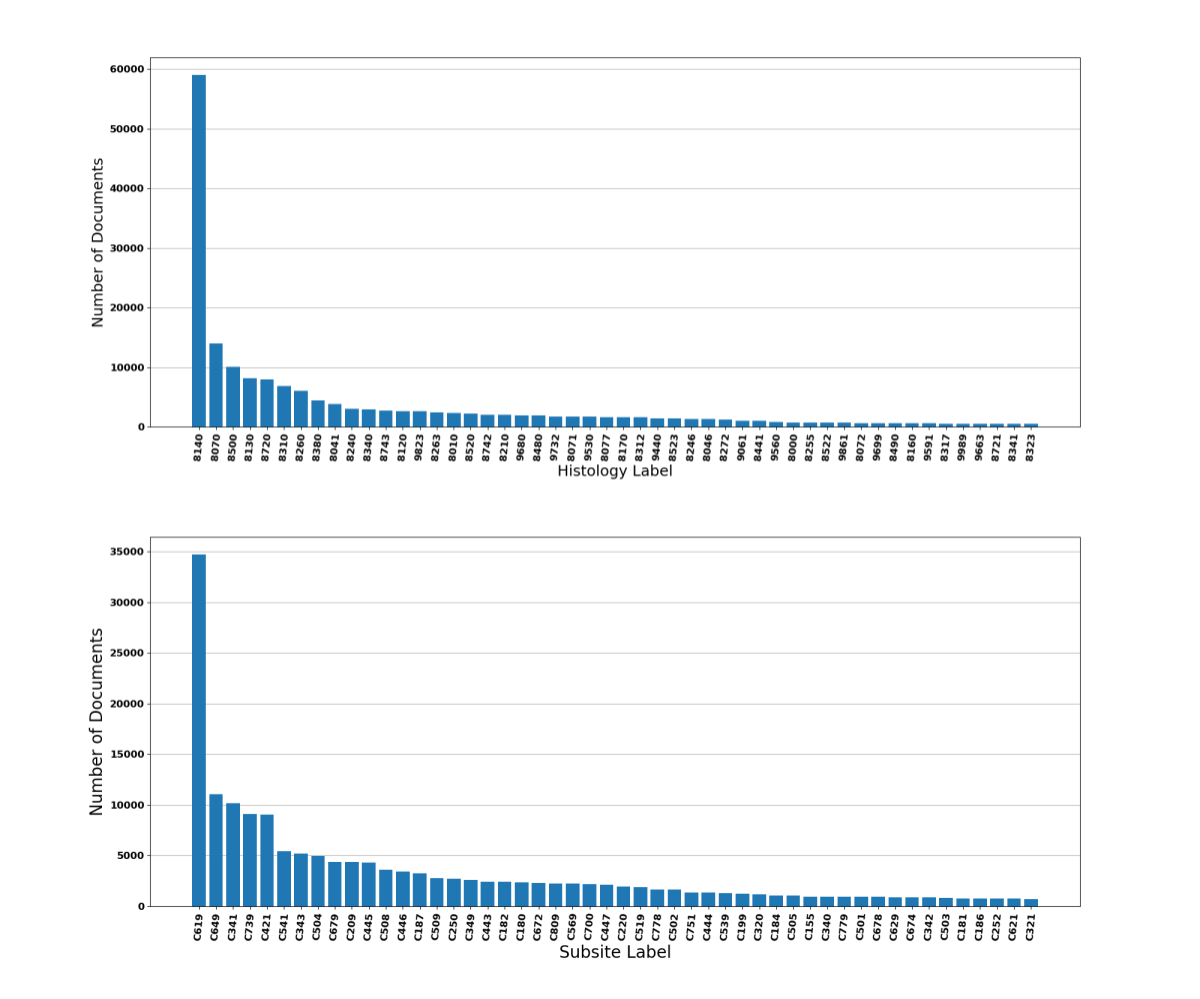

Supplement: Supplementary file 1 — Additional file 1. Dataset class imbalance plots. [file 12859_2021_4047_MOESM1_ESM.tif]

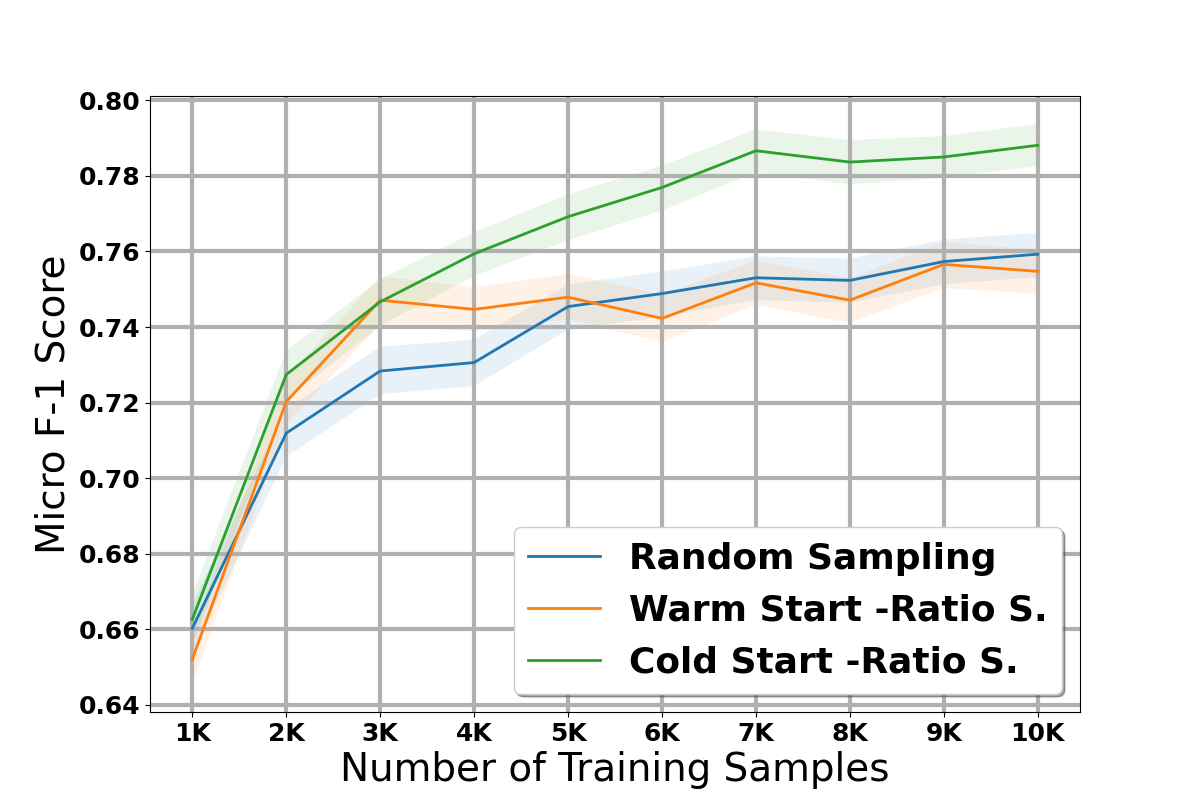

Supplement: Supplementary file 4 — Additional file 4. Performance of cold start ratio sampling, warm start ratio sampling, and random sampling on the histology task (small dataset). [file 12859_2021_4047_MOESM4_ESM.tif]

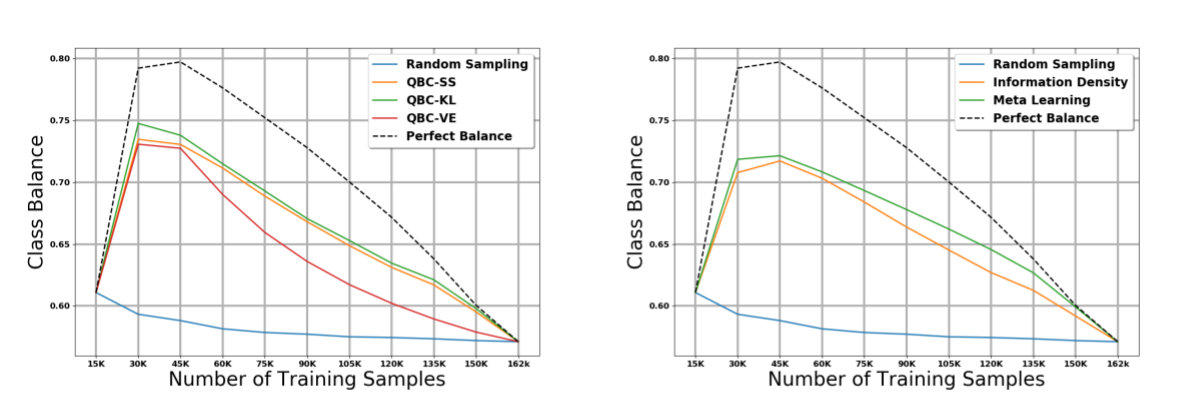

Supplement: Supplementary file 10 — Additional file 10. Large dataset: class imbalance - histology task. [file 12859_2021_4047_MOESM10_ESM.tif]

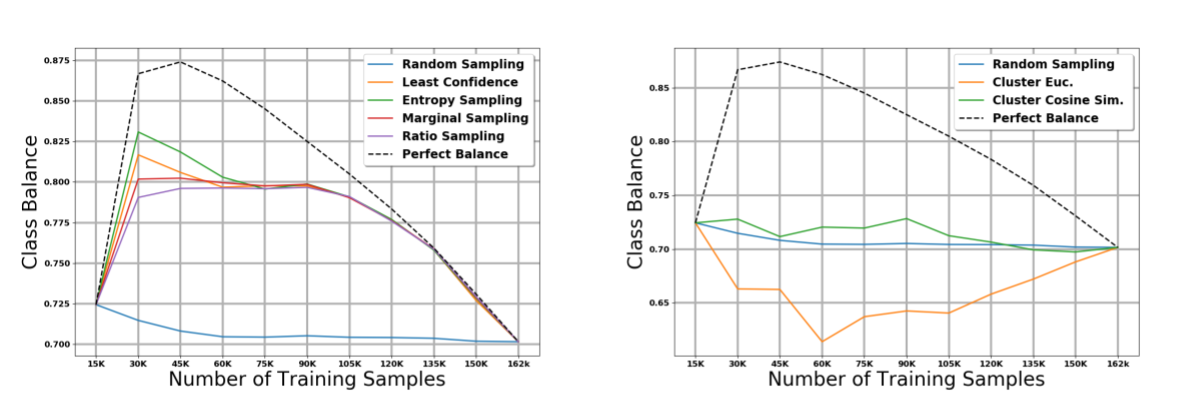

Supplement: Supplementary file 11 — Additional file 11. Large dataset: class imbalance - subsite task. [file 12859_2021_4047_MOESM11_ESM.tif]

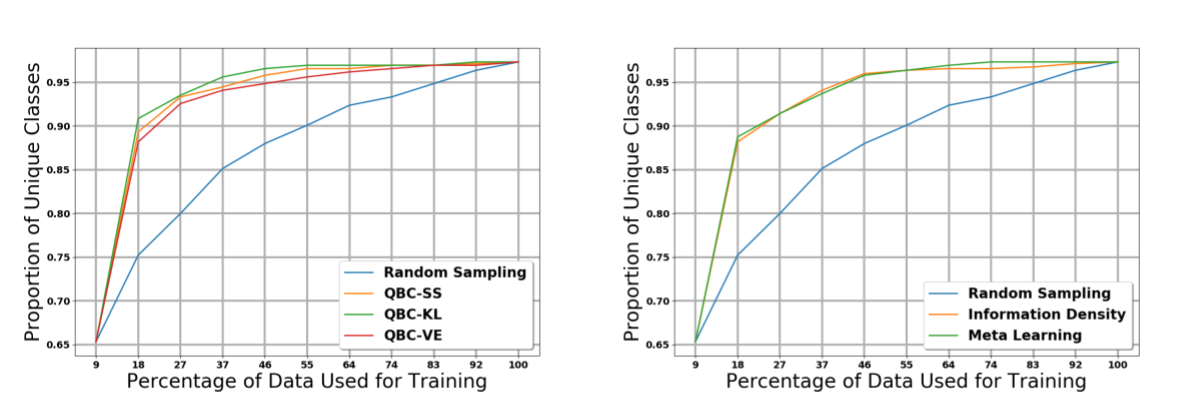

Supplement: Supplementary file 12 — Additional file 12. Small dataset: class imbalance - histology task. [file 12859_2021_4047_MOESM12_ESM.tif]

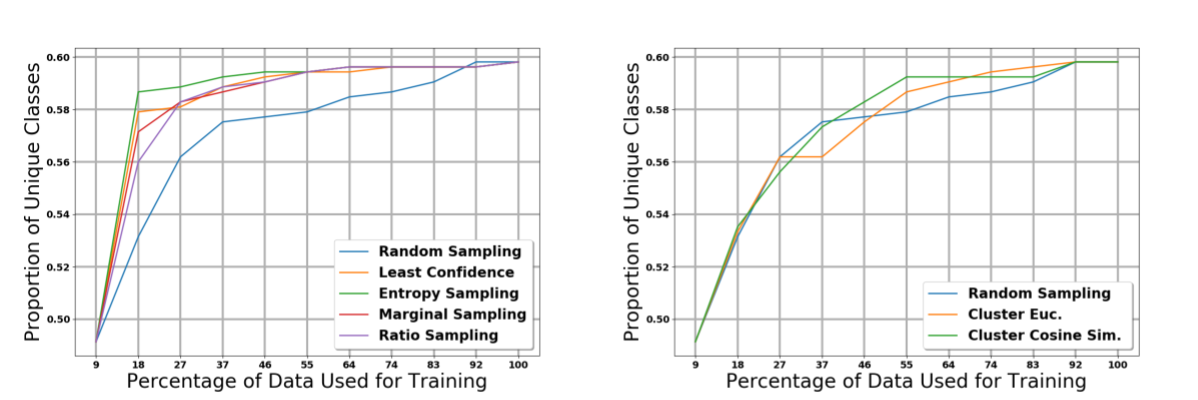

Supplement: Supplementary file 13 — Additional file 13. Small dataset: class imbalance - subsite task. [file 12859_2021_4047_MOESM13_ESM.tif]

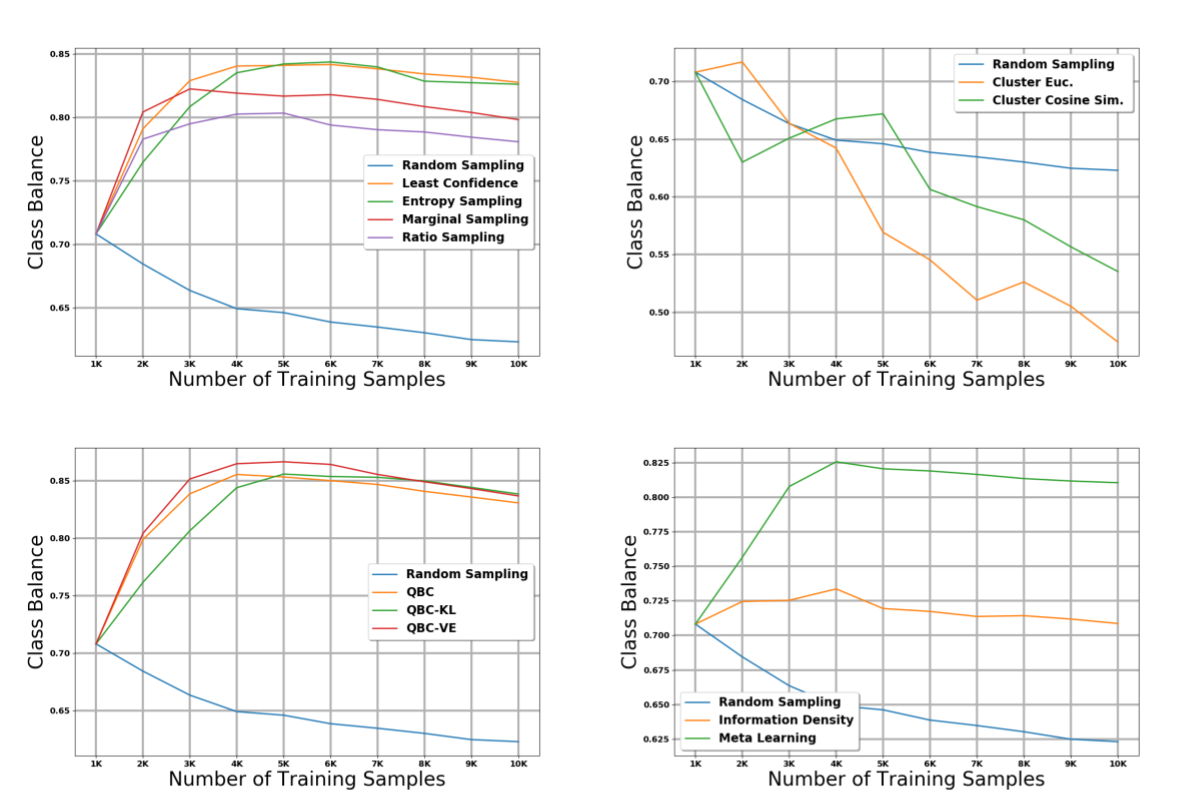

Supplement: Supplementary file 14 — Additional file 14. Large dataset: class proportion plots - histology. [file 12859_2021_4047_MOESM14_ESM.tif]

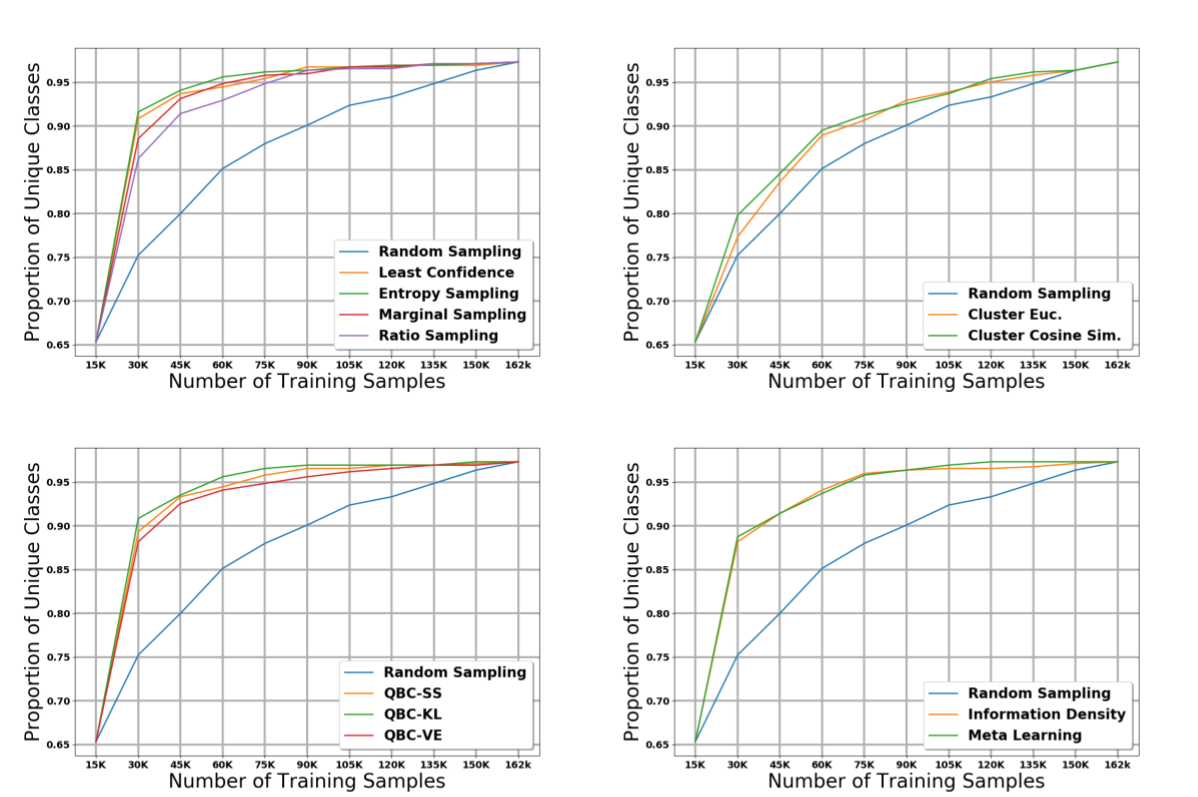

Supplement: Supplementary file 15 — Additional file 15. Large dataset: class proportion plots - subsite task. [file 12859_2021_4047_MOESM15_ESM.tif]

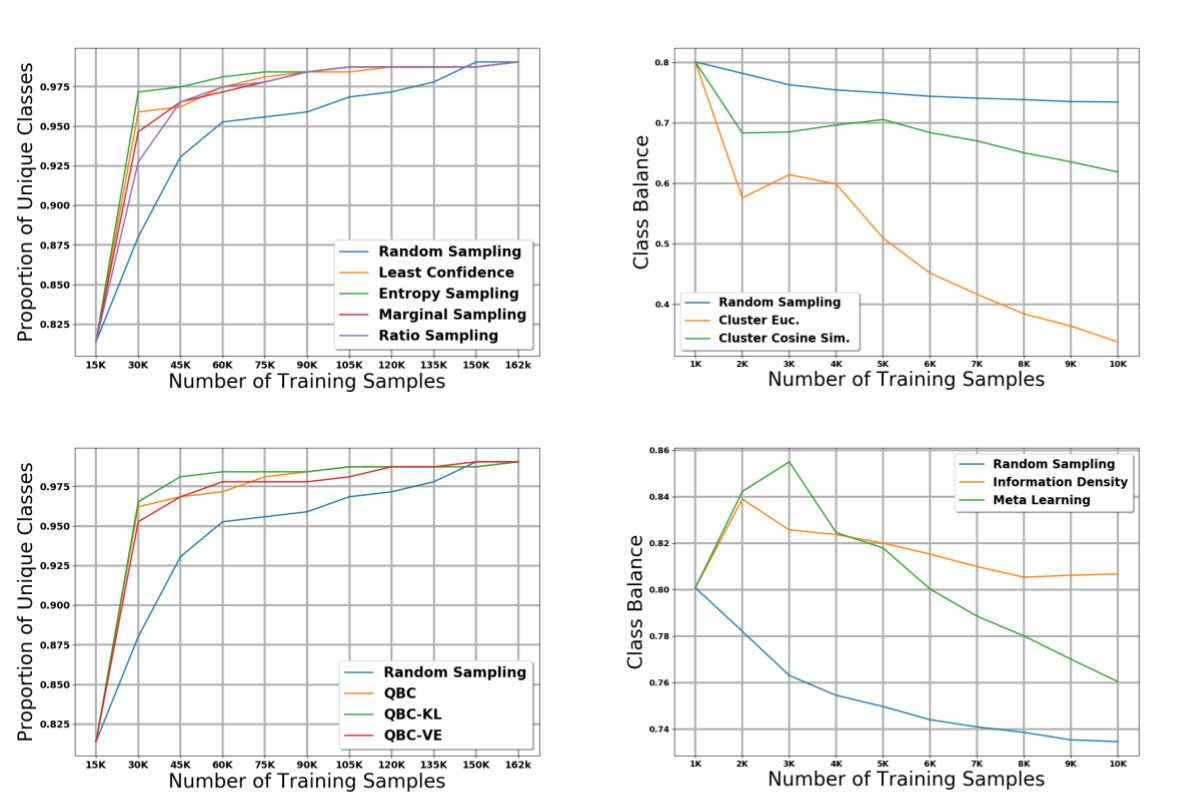

Supplement: Supplementary file 16 — Additional file 16. Small dataset: class proportion plots - histology task. [file 12859_2021_4047_MOESM16_ESM.tif]

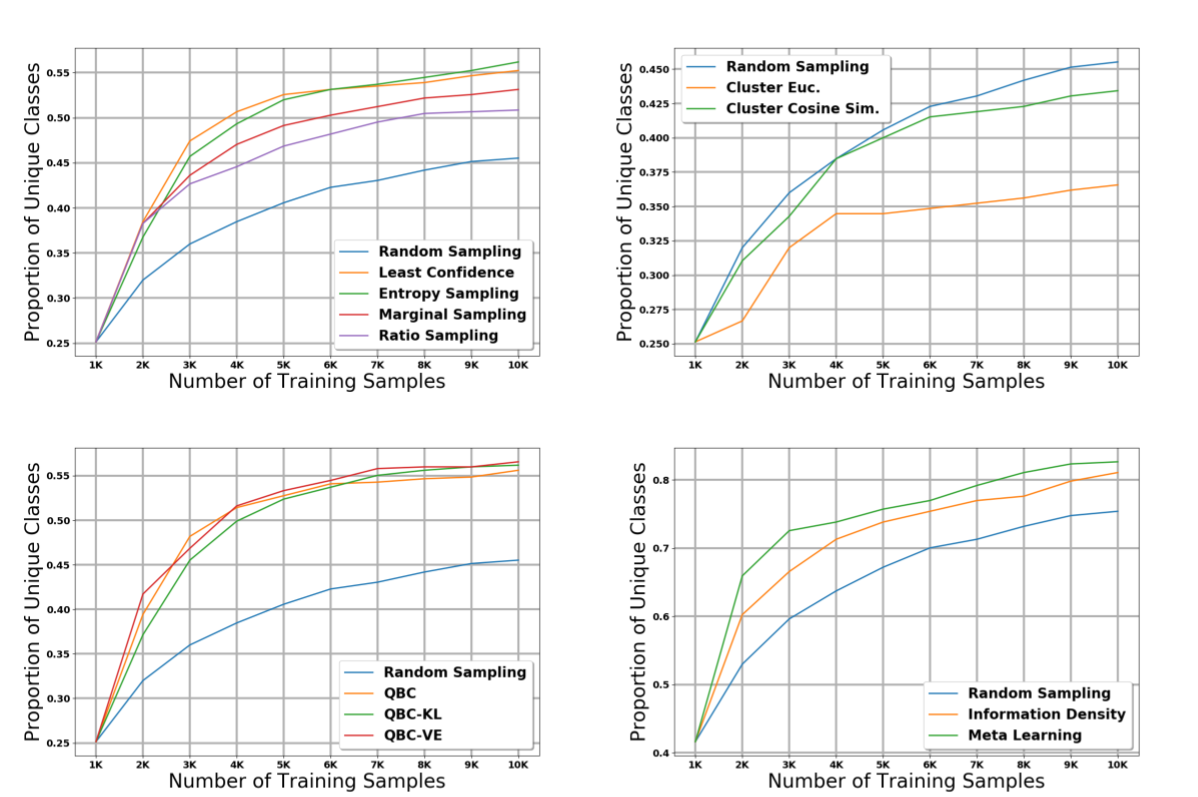

Supplement: Supplementary file 17 — Additional file 17. Small dataset: class proportion plots - subsite task. [file 12859_2021_4047_MOESM17_ESM.tif]

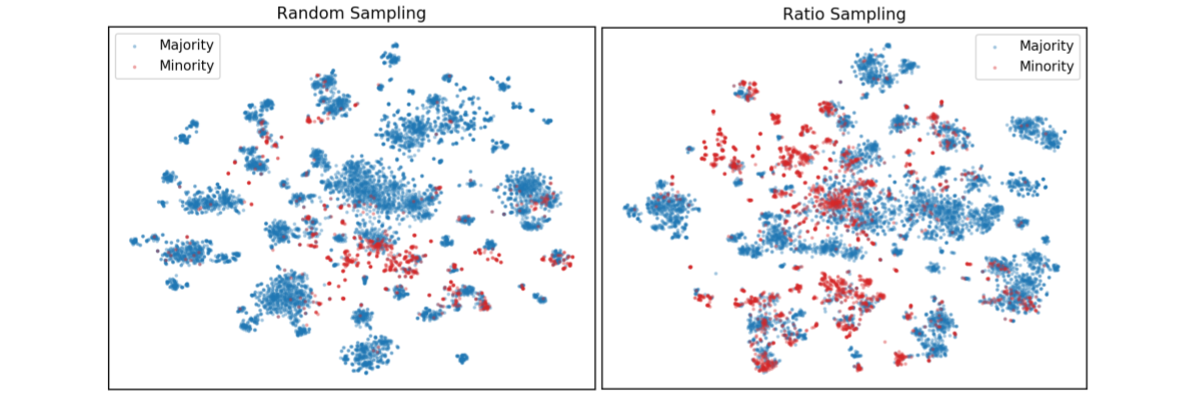

Supplement: Supplementary file 18 — Additional file 18. Document embeddings generated via TSNE for histology task (small dataset) with and without10 iterations of active learning. Documents are colored by majority class (number of total samples in dataset aboveaverage) and minority class (number of total samples in dataset below average). [file 12859_2021_4047_MOESM18_ESM.tif]
