# Supplementary material for: Deep active learning for classifying cancer pathology reports
Source: BMC Bioinformatics. 2021 Mar 9;22:113. doi: 10.1186/s12859-021-04047-1 (PMC7941989; doi:10.1186/s12859-021-04047-1)
Supplement: Supplementary file 2 — Additional file 2. Text preprocessing steps. [file 12859_2021_4047_MOESM2_ESM.docx]

Text Preprocessing Steps

1. Remove identifier segments (registry ID, patient ID, tumor number, and document ID)
2. Remove XML tags
3. Convert unicode to ASCII
4. Lowercase
5. Replace tabs and line breaks with spaces
6. Replacing all instances of floats with the string “floattoken”
7. Replace all integers higher than 100 with the string “largeinttoken” (to reduce the number of unique tokens associated with numbers)
8. If the same non-alphanumeric character appears consecutively more than once, replace it with a single copy of that character
9. Add a space before and after every non-alphanumeric character
10. Remove words longer than 25 characters to reduce noise (these are generally artifacts from format conversions)
11. Tokenize document
12. Replace any token that appears less than 5 times across the entire corpus with the string “unknowntoken”
13. Add padding or truncate document to 1500 tokens
