# Supplementary material for: Deep active learning for classifying cancer pathology reports
Source: BMC Bioinformatics. 2021 Mar 9;22:113. doi: 10.1186/s12859-021-04047-1 (PMC7941989; doi:10.1186/s12859-021-04047-1)
Supplement: Supplementary file 3 — Additional file 3. Bootstrapping procedure for confidence. [file 12859_2021_4047_MOESM3_ESM.docx]

Bootstrapping procedure for confidence

The 95% confidence intervals presented in this paper were computed as follows:

- Compute and save the accuracy of every model on the test data set; this is the original accuracy.
- Create a new dataset by sampling documents with replacement from the test dataset.
- Find the accuracy of the model in this new dataset.
- Repeat steps two and three 2,000 times and store all accuracy results.
- Calculate the 95\% confidence interval by finding the 2.5 and 97.5 percentiles.
